# The Evolutionary Plasticity, Conservation of Functional Motifs, and Structural—Functional Architecture of the *ras85D* 3′ UTR in *Drosophila*

**DOI:** 10.3390/genes17091041

**Published:** 2026-08-29

**Authors:** Aleksey M. Kulikov, Ekaterina A. Sivoplyas, Oleg E. Lazebny

**Affiliations:** 1 N.K. Koltzov Institute of Developmental Biology, Russian Academy of Sciences, Moscow 119334, Russia; sivoplyas-ekater@mail.ru (E.A.S.); o.e.lazebny@idbras.ru (O.E.L.); 2 Campus of Biology and Chemistry, Department of Biochemistry, Molecular Biology, and Genetics, Moscow Pedagogical State University, Moscow 119991, Russia

**Keywords:** 3′ UTR, *ras85D*, *Drosophila*, alternative polyadenylation, insertions and deletions, evolutionarily conserved motifs, RNA secondary structure, boundary interface, miRNA

## Abstract

**Background/Objectives:** The 3′ untranslated region (3′ UTR) integrates cleavage and polyadenylation signals, microRNA targets, RNA-binding-protein sites, and RNA secondary structure, but the organizational levels that remain conserved during long-term sequence evolution are poorly understood. **Methods:** We analyzed the *ras85D* 3′ UTR in 37 drosophilid taxa. Substitution rates were estimated by maximum likelihood and RelTime; insertions and deletions were reconstructed with ARPIP and summarized as insertion–deletion evolutionary localizations (IELs). Mobile-element candidates were detected with CENSOR/Repbase, and evolutionarily conserved motifs (ECMs) with MEME/MAST. Functional and structural annotations were integrated for *Drosophila melanogaster*, *Drosophila yakuba*, and *Drosophila virilis* and tested using permutation-based coverage, distance, boundary-neighborhood, and multilayer architecture analyses. **Results:** The 2247-column alignment yielded 959 block events (710 deletions and 249 insertions). Among 63 positive-length ingroup branches, 16 were deletion-enriched, three were insertion-enriched, and one showed bidirectional turnover. Thirty-four IELs projected to 27 *D. melanogaster* loci and were associated with ECMs. The final registry contained 390 primary functional objects and 1135 RNAfold-predicted structural segments. Predicted weakly conserved miRNA target sites were depleted in ECM_15 and ECM_11, whereas none of 12 Functional Distance tests was significant. APA objects were enriched near predicted structural-segment boundaries (O/E = 5.21; FDR = 0.00761). SAME_MULTILOOP_INTERVAL showed reduced between-context variance (0.276× null; FDR = 0.0233), and the DIFFERENT_MULTILOOP_ARMS − SAME_MULTILOOP_INTERVAL contrast was significant (*p* = 0.00149; FDR = 0.00447). **Conclusions:** The *ras85D* 3′ UTR evolves as a mosaic system in which extensive deletion-biased sequence turnover coexists with conserved regulatory landmarks, recurrently remodeled local neighborhoods, and context-dependent structural–functional architectures.

## 1. Introduction

The 3′ untranslated regions (3′ UTRs) are integrative regulatory regions of mRNAs that influence transcript stability and localization, translation, RNA decay, and the selection of alternative 3′ ends. These functions arise from combinations of cleavage and polyadenylation sites, microRNA targets, RNA-binding-protein sites, and local RNA secondary structure [1,2,3,4]. Thus, 3′ UTR evolution cannot be described solely by nucleotide-substitution rates: changes in length, gain and loss of regulatory motifs, remodeling of distances between them, and altered structural context are also important.

Alternative polyadenylation (APA) generates isoforms with different 3′ UTR lengths and consequently changes the accessible set of cis-regulatory elements. Alternative 3′ ends are widespread in eukaryotic genes, and their usage depends on tissue, developmental stage, and physiological state [3,5,6]. In *Drosophila*, robust tissue-level trends include 3′ UTR lengthening in the nervous system and preferential use of shorter isoforms in the male germline. Interspecific differences may reflect not only newly acquired sites but also changes in PAS and DSE sequence and position relative to the cleavage site [4].

A canonical APA module contains upstream UGUA elements and PAS hexamers, the cleavage site itself, and downstream GU- or U-rich elements (DSEs). These elements are recognized by CPSF, CstF, CFIm, CFIIm, and other RNA-binding proteins. Cleavage-site efficiency depends on motif sequence, spacing, and accessibility. Although empirical strength scales have been developed for vertebrate PAS variants, the exact hierarchy of hexamers in invertebrates, including *Drosophila*, remains insufficiently defined. APA-architecture analysis should therefore separate effects of element presence and spacing from assumptions about the intrinsic strength of a particular hexamer.

MicroRNA targets form a second major layer of 3′ UTR architecture. Their functional impact depends not only on seed complementarity but also on transcript position, local sequence composition, accessibility, and the availability of alternative isoforms [7,8]. A change in 3′ UTR length can therefore alter APA and the accessible miRNA target repertoire simultaneously. Evolutionarily conserved motifs may be maintained because they integrate several regulatory functions or act as stable landmarks between more plastic sequence segments.

RNA secondary structure can alter the effective proximity and accessibility of motifs, but its role cannot be reduced to visual distance in a two-dimensional drawing. Formal analysis must distinguish local structural classes, boundaries and centers of structural segments, and multilayer relationships among RNA structure, APA, ECMs, and evolutionarily remodeled regions. These properties describe RNA architecture but should not be interpreted as direct three-dimensional physical contacts.

The *ras85D* gene provides a suitable model for this analysis. Its protein product belongs to the highly conserved Ras family of small GTPases and transmits signals from receptor tyrosine kinases to MAPK pathways controlling cell division, differentiation, adhesion, and motility [9,10,11,12]. Despite strong conservation of the coding region, *ras85D* non-coding regions show substantial interspecific variability. The transcript also has multiple tissue- and stage-specific 3′ ends, allowing sequence evolution to be linked to the functional architecture of APA.

The aim of this study was to construct a coordinated multilayer model of *ras85D* 3′ UTR evolution. At the broad phylogenetic level, we analyzed point substitutions, insertions, deletions, microsatellite variation, and candidate mobile-element traces in 37 taxa. At the detailed level, conservation of ECMs, miRNA targets and APA components, turnover of immediate boundary neighborhoods, and RNA secondary structure were examined in *D. melanogaster*, *D. yakuba*, and *D. virilis*. We separately tested functional-object coverage, distances to PAS and cleavage sites, multilayer architectural relationships, and the interspecific consensus of architectural domains.

## 2. Materials and Methods

The analytical framework was organized as a sequence of linked but partly independent layers. First, the 37-taxon alignment was used for phylogenetic and evolutionary-rate analyses and for ARPIP reconstruction of indels; recurrently remodeled alignment intervals were then summarized as IELs. Second, evolutionarily conserved motifs (ECMs) were identified across the broader taxon set, whereas APA components, miRNA target-site annotations, and RNA secondary-structure models were integrated in detail for *D. melanogaster*, *D. yakuba*, and *D. virilis*. Third, relationships among these layers were tested with boundary-interface, coverage/distance, between-context dispersion, and Pairwise Architecture Test (PAT) analyses. Finally, the Architecture Evidence Matrix (AEM) and Basal Architecture Network (BAN) integrated evidence across IEL, ECM, APA, and RNA-structure layers. Thus, the PAT provides pairwise statistical tests between annotation layers, whereas the AEM and BAN summarize their multilayer architectural relationships.

### 2.1. Sequences and Multiple Alignment

The final phylogenetic dataset comprised 37 drosophilid taxa, with *Scaptodrosophila lebanonensis* (*S. lebanonensis*) used as the outgroup. Orthologous *ras85D* 3′ UTR sequences were obtained from NCBI Genome Datasets and previously assembled species-specific genomes. The complete taxon list, sources, coordinates, and mapping evidence are provided in Supplementary Methods SM1, Table S1, and Data S1.

Multiple alignments were generated with ClustalW and MUSCLE [13,14] and manually inspected in regions containing long indels and microsatellites. After final curation, the alignment comprised 2247 columns. The reference *D. melanogaster* 3′ UTR was 964 nt long; the *D. yakuba* and *D. virilis* sequences used for the detailed three-species analysis were 968 and 1150 nt, respectively. Functional-object coordinates were stored as 1-based inclusive intervals; species-specific and alignment coordinates were retained in parallel for interspecific comparisons.

### 2.2. Nucleotide-Substitution and Evolutionary-Rate Analyses

Phylogenetic analyses were performed in MEGA 12 [15] using maximum likelihood. Sensitivity to gap filtering was evaluated with two matrices: Complete Deletion, containing 391 gap-free positions, and Partial Deletion with a 10% site-coverage threshold, containing 1530 positions. Models were selected using BIC and AICc. T92+G+I [16] was used for both matrices; for the Complete Deletion matrix, the estimated proportion of invariant sites was zero. The strict molecular-clock hypothesis was tested by comparing log-likelihoods of models with and without the equal-rate constraint.

Divergence times and lineage-specific relative rates were estimated with RelTime [17,18], without assuming a single global rate. Calibrations were based on published multilocus and fossil-informed estimates. The divergence between the *Drosophila* and *Sophophora* subgenera was constrained to 40–45 Ma [19,20]. Within *Sophophora*, the adopted ranges were 0.25–0.30 Ma for *Drosophila simulans*–*Drosophila sechellia*, 2.5–3 Ma for *D. melanogaster*–*D. simulans*, 10–12 Ma for *D. melanogaster*–*Drosophila erecta*, and 18–22 Ma for *D. melanogaster*–*Drosophila ananassae* [20,21]. Within the *Drosophila subgenus*, calibrations included 2.5–3 Ma for *D. virilis*–*Drosophila lummei*, 3–4 Ma for *Drosophila montana*–*Drosophila lacicola*, and 7–10 Ma for the *D. virilis* and *D. montana* clades [22,23,24].

Two rate measures were analyzed for the 36 terminal ingroup taxa. Terminal relative rate was taken directly from the Rate field of the exported RelTime tree. The cumulative mean root-to-tip rate was calculated as the sum of maximum-likelihood branch lengths along the root-to-tip path divided by the calibrated root age. Distributions were summarized by empirical observations, the median, interquartile range, and full range without assuming normality. The two gap-filtering strategies were compared with the paired Wilcoxon test; rank and linear agreement were assessed with Spearman and Pearson coefficients, respectively. Rates derived for individual young terminal edges were used only for sensitivity analyses because division by very small node ages makes them unstable.

The likelihood-ratio statistic for the strict-clock test was calculated as 2ΔlnL = 2(lnL_no-clock − lnL_clock), with degrees of freedom defined by the difference in model parameters. Full model parameters, taxon-specific rates, and sensitivity analyses are provided in Supplementary Methods SM2, Table S2, Figure S1, and Script S1.

### 2.3. Reconstruction of Insertions and Deletions

Phylogenetic placement of insertions and deletions was performed with ARPIP, which reconstructs ancestral sequences under the Poisson Indel Process [25]. ARPIP and the Bio++ dependencies were compiled from source in Google Colab. The input alignment, Newick tree, configuration, execution log, executable, and complete software environment were retained for reproducibility.

ARPIP output contained events at individual alignment columns. Adjacent events of the same type assigned to the same branch and forming a continuous interval were merged into a single block event. Event type, branch, alignment coordinates, and length were recorded. Branch-intensity analyses included 63 positive-length branches.

For each branch, expected insertion and deletion counts were calculated in proportion to branch length and the total number of events of the corresponding type. Departure from expectation was described by the observed-to-expected ratio (O/E), and *p*-values were adjusted with the Benjamini–Hochberg procedure. Branches were classified as insertion-enriched, deletion-enriched, bidirectional-turnover, or non-significant.

The dependence of event count on phylogenetic position was analyzed by negative-binomial regression using log branch length as the exposure. Predictors were normalized root distance and an internal-branch indicator. Exponentiated coefficients were reported as rate ratios. Internal and terminal branches were additionally compared with the Mann–Whitney test. The canonical 63-branch registry, branch regimes, and reproducibility materials are provided in Supplementary Methods SM3, Table S4, Data S2, and Script S1.

### 2.4. Definition of IELs and Spatial Permutation Tests

Reconstructed indel events were grouped into insertion–deletion evolutionary localizations (IELs), alignment regions in which recurrent events assigned to different branches formed a single evolutionarily heterogeneous region. IELs were not treated as individual mutations; they served as integrative units of spatial analysis. IEL coordinates were projected onto the *D. melanogaster* 3′ UTR and classified as direct, partially gapped, or between-nucleotide mappings. Multiple evolutionary IELs projecting to the same reference interval were collapsed into a unique *D. melanogaster* locus for the primary model, whereas all 34 IELs were retained separately in sensitivity analyses.

IEL overlap with ECMs and ECM proximity to IEL boundaries were evaluated by interval-preserving permutations. The number, lengths, and internal configuration of the tested intervals were preserved while their positions relative to the fixed ECM map were changed. A stricter sensitivity analysis used circular shifts, in which all intervals received the same shift modulo sequence length; this null model preserves interval lengths, pairwise distances, clustering, and autocorrelation [26,27]. Individual analyses used up to 100,000 permutations, and *p*-values were computed with a +1 correction in the numerator and denominator.

Empirical *p*-values were calculated as *p* = (b + 1)/(B + 1), where B is the number of permutations and b is the number of permuted statistics at least as extreme as the observed statistic. This correction prevents zero permutation *p*-values and follows the recommendation of Phipson and Smyth [28].

In the strict spatial null model, all intervals in one coordinate track were shifted by the same k modulo sequence length L: x′ = ((x − 1 + k) mod L) + 1. The second object map remained fixed, so the relative phase of the two maps changed while interval number and length, pairwise distances, local clustering, and spatial autocorrelation of the shifted track were preserved. This is an adaptation of circular genomic permutation to a single 3′ UTR sequence [27].

Multiple comparisons were controlled by the Benjamini–Hochberg false discovery rate procedure [29]. Correction was performed separately within each predefined family of hypotheses. IEL definitions, coordinate projections, and spatial tests are documented in Supplementary Methods SM4, Table S5, Figure S3, and Data S2.

### 2.5. Microsatellites and Mobile Elements

Microsatellite and low-complexity regions were identified in the multiple alignment from repeated di- and mononucleotide motifs. Associations between 3′ UTR length and the number of alignment-derived indel differences were evaluated with Pearson and Spearman coefficients. This analysis described present-day length and difference counts and was not used to infer the direction of individual ancestral events.

Mobile-element homology was searched with CENSOR using the GIRI Repbase library and a *Drosophila filter* [30]. A candidate transposon insertion was accepted when high sequence similarity and alignment score coincided with phylogenetic specificity of the interval. Orientation, coordinates, similarity, and position relative to ECMs were recorded separately. The catalogues of mobile-element candidates, microsatellite context, and ECMs are provided in Supplementary Methods SM5, Table S3, and Data S3.

### 2.6. ECM Discovery and Functional Annotation

Evolutionarily conserved motifs (ECMs) were identified with MEME Suite 5.5.9 [31,32]. MEME searches were performed on DNA under the ZOOPS model, allowing both strands, with -nmotifs 17, -minw 6, -maxw 30, -minsites 15, -maxsites 40, -revcomp, and -markov_order 0. MAST was then used to map the complete 17-motif set to each species-specific unaligned 3′ UTR [33]. Because motif positions are reported in the coordinate system of each individual sequence, the horizontal positions in the MAST map are not a shared interspecific coordinate scale. MAST combined *p*-values describe support for the full motif signature and were not interpreted as functional *p*-values for individual motif occurrences.

Potential miRNA targets were annotated with TargetScanFly 7.2 [8]. Coordinate lists were prioritized over the Total field; multiple positions of the same family were treated as separate 7-mer sites. Exact duplicates were removed and recorded in the QC log. PAS, UGUA, DSE, and cleavage sites in *D. melanogaster*, *D. yakuba*, and *D. virilis* were coordinated using published APA datasets [4,6] and verified directly in the sequences. The final agreed registry, inventories, and coordinate rules are provided in Supplementary Methods SM6, Table S6, and Data S3.

The evidence status of the annotation layers differed. Cleavage-site positions and usage were derived from published experimental 3′-end datasets, whereas individual PAS, UGUA, and DSE components were sequence annotations coordinated to those data. Potential miRNA target sites were computational predictions from TargetScanFly, and ECMs were computationally identified sequence-conservation motifs from MEME/MAST. RNA secondary-structure features analyzed below were likewise computational predictions. Conservation, overlap, proximity, or statistical association of a predicted feature was therefore not treated as an experimental demonstration of biological function.

### 2.7. Secondary Structure and Structural Graphs

MFE and centroid structures were modeled for *D. melanogaster*, *D. yakuba*, and *D. virilis* with the RNAfold web server [34,35]. CT and dot–bracket representations were cross-checked. Each nucleotide was assigned to one of six local structural classes: stem, hairpin loop, internal loop, bulge, multiloop, or exterior.

Two graph levels were constructed. The nucleotide graph included sequence-adjacency and complementary base-pairing edges. The structural-element graph grouped nucleotides belonging to the same structural segment and described their topological relationships. MFE and centroid models were analyzed separately and then combined in sensitivity analyses.

For each structural segment, model, class, species-specific coordinates, and alignment coordinates were recorded. Subsequent analyses tested coverage of individual structural classes, positions of functional objects relative to segment boundaries and centers, and multilayer relationships of structure with APA and ECMs. Structural annotations were treated as computational models of local organization rather than direct three-dimensional distances.

### 2.8. Interspecific Object Registry and Evolutionary Groups

Functional and structural annotations from the three species were combined into one coordinate registry. After obsolete legacy assignments were excluded, the final set contained 1525 objects: 390 primary functional objects and 1135 structural segments. Functional objects were grouped into evolutionary families according to homologous alignment coordinates, object type, and sequence evidence. Missing states were classified explicitly as true absence, unresolved, or not applicable. A full registry summary is provided in Supplementary Methods SM6, Table S6, and Data S3. Throughout the manuscript, “functional object” is used as an operational registry category for annotated sequence features and does not imply experimental validation of every individual object.

### 2.9. Boundary Interfaces and Their Evolutionary Conservation

A boundary interface was defined as the immediate linear coordinate neighborhood of two annotated 3′ UTR objects in the 5′→3′ direction. The term was used exclusively for coordinate adjacency and did not imply a spatial or molecular interaction. Two configurations were distinguished: SHARED_COORDINATE, in which adjacent objects shared a boundary coordinate, and ABUTTING_CUT, in which the end of the upstream object was immediately followed by the start of the downstream object.

A directed graph of strict functional interfaces was constructed for each species. Interfaces between homologous object groups were merged into evolutionary families. For each family, species presence, immediate partners, boundary-neighborhood configuration, and lineage-specific gains or losses were recorded.

General preferences among object-type pairs were tested by conditional randomization within the Boundary Pair Universe. For each permutation, eligible endpoint pairs were sampled without replacement while preserving the observed number of interfaces within matching geometric strata defined by species, homotypy, layer relation, interval relation, gap bin, span bin, and endpoint-length-pair bin. The Boundary Pair Universe, species states, and analysis code are documented in Supplementary Methods SM7, Table S7, Data S3, and Script S3. Two normalized historical input CSV files used to construct the original BPU were not retained after working-archive cleanup; the original builder and randomization scripts, recorded input checksums, QC records, and compact final outputs are publicly archived, and no later registry was substituted for the missing inputs.

### 2.10. Coverage Enrichment and Functional Distance

Both analyses used the final agreed set of 510 *D. melanogaster* objects within 3′ UTR coordinates 1–964. Coverage Enrichment compared the fraction of nucleotides covered by functional classes across structural classes, individual ECMs, pooled ECMs, and APA-centered windows. For PAS, cleavage sites, and the pooled APA layer, proximal ±25 nt windows and corresponding distal control regions were used.

In the coverage null model, overlapping or adjacent observed feature intervals were first merged. The number and multiset of merged-interval lengths were then preserved, and each interval was independently relocated uniformly within the 964 nt 3′ UTR; randomized placements were allowed to overlap, whereas tested and control region masks remained fixed. Each comparison used 10,000 permutations with seed 20260718. BH/FDR correction was applied within predefined species × test-family × feature families.

Functional Distance measured the median minimum edge-to-edge distance from functional intervals to fixed PAS and cleavage sites. Self-comparisons were excluded; null distributions used the same length-preserving interval-relocation scheme while PAS and cleavage intervals remained fixed. Full parameters and results are provided in Supplementary Methods SM8, Table S9, Data S4, and Script S4.

For descriptive visualization, annotated APA elements were additionally combined into nine coordinate clusters (C001–C009) based on overlapping intervals and shared components. This grouping was used only for descriptive visualization; the final coverage/distance and PAT analyses were calculated from individual objects and regions in the final agreed annotation and did not use cluster membership as a statistical unit.

### 2.11. Between-Context Dispersion of Cleavage-Site Usage

Absolute isoform counts and usage fractions were available for 29 cleavage sites in five comparable contexts: head, ovary, testis, embryo 0–12 h, and embryo 12–24 h. Final five-component structural profiles were available for 27 sites. Usage fractions were transformed to empirical logits with a 0.5 continuity correction. To separate tissue- and stage-dependent changes from differences in mean site strength, each profile was centered on the five-context mean of that cleavage site.

For each of five mutually exclusive states, weighted variance of centered values was calculated; the weight was the consensus fraction of that state across centroid and MFE annotations of all assigned APA-component pairs. In the null model, complete five-component profiles were permuted among sites within species while the five linked observations for each site remained together. The prespecified primary contrast was DIFFERENT_MULTILOOP_ARMS − SAME_MULTILOOP_INTERVAL. We used 100,000 permutations with seed 20260727 and analyzed all five contexts, the three adult tissues, and the two embryonic stages separately.

Robustness was evaluated with centroid and MFE models separately, with component-only pairs, with pairs involving a cleavage site, by species, and by leave-one-species-out analyses. Because the source data contained one aggregated value for each species × tissue/stage combination, the analysis quantifies between-context heterogeneity rather than biological-replicate variance within tissues. Full results are provided in Supplementary Methods SM10, Table S10, Figure S5, Data S5, and Script S5.

### 2.12. Pairwise Architecture Test, Architecture Evidence Matrix, and BAN

The Pairwise Architecture Test (PAT) evaluated three multilayer edges: ECM–predicted RNA structure, ECM–APA, and predicted RNA structure–APA. The final agreed input contained 49 ECM occurrences, 127 APA objects, and 1135 RNAfold-predicted structural segments in an alignment3 coordinate system of 1315 columns. The null model was the complete set of 1314 non-zero circular shifts of one layer relative to the other; FDR was calculated separately within each edge and metric family.

The Architecture Evidence Matrix (AEM) integrated ECM presence, APA proximity, and overlap with structural segments for each of 34 IELs in *D. melanogaster*, *D. yakuba*, and *D. virilis* and then assigned an interspecific consensus architectural class. The Basal Architecture Network (BAN) represented the evidence network among IEL, ECM, RNA-structure, and APA layers; edge grade reflected evidence source and statistical support and did not imply direct physical contact. Details are provided in Supplementary Methods SM9, Table S8, Figure S4, Data S4, and Script S4. The hierarchy of datasets and statistical models used in this study is summarized in Table 1.

### 2.13. Statistical Analysis and Computational Implementation

Standard and specialized analyses were performed in R 4.2.1 [36], using RStudio 41.7.2 as the interface, and Python 3.13.5. The fitdistrplus package [37] was used during an early exploratory stage to compare theoretical rate distributions; the final analysis did not assume Gaussian distributions and reported empirical distributions, medians, and interquartile ranges. Coordinate, interval, permutation, graph, and tissue-specific analyses were implemented in custom Python scripts.

Numerical arrays were processed with NumPy 2.3.5 [38], tabular data with pandas 2.2.3 [39], statistical functions and numerical optimization with SciPy 1.17.0 [40], and graph construction and analysis with NetworkX 3.6.1 [41].

Computations were performed in Linux using Python scripts and interactive Jupyter/Google Colaboratory notebooks [42]. Fixed pseudorandom-number seeds were used for every stochastic procedure; permutation counts and seeds are given in the relevant method descriptions and Supplementary Tables. Manuscript-associated custom scripts, final analysis inputs, machine-readable outputs, QC records, and computational-environment information are publicly available at https://github.com/alexgenego/ras85d-3utr-architecture (accessed on 26 August 2026). The fixed release corresponding to this revision (v1.0.0) is permanently archived in Zenodo at https://doi.org/10.5281/zenodo.22108845. Known provenance limitations, including the two missing historical BPU input files, are documented in the repository.

GenAI was used to assist with writing and validating Python code, organizing the reproducibility archive, and supporting quality control, including completion of the QC log. All analyses were executed by the authors, and the resulting outputs were reviewed against the underlying data and predefined analysis specifications.

## 3. Results

### 3.1. Overall Sequence Variation and Nucleotide-Substitution Rates

The *D. melanogaster* 3′ UTR was 964 nt long, whereas sequences from the other taxa ranged approximately from 798 to 1443 nt. The final multiple alignment contained 2247 columns, 1283 of which corresponded to gaps in the *D. melanogaster* reference. The large expansion of alignment length relative to individual sequences indicates a substantial contribution of insertion–deletion variation.

Mean nucleotide identity in the initial comparative analysis was 76.5%. The sequences were AT-rich: mean A and T fractions were 31.9% and 34.3%, whereas C and G fractions were 16.9% and 17.0%. The transition/transversion ratio was 0.676. Thus, despite common ancestry, the locus combines conserved homologous segments with pronounced length and composition variability in individual intervals.

The strict molecular-clock hypothesis was rejected under both alignment-filtering strategies. For the 391-position Complete Deletion matrix, the unconstrained model had a significantly higher likelihood than the strict-clock model (2ΔlnL = 116.404; df = 35; *p* = 2.10 × 10^−10^). For the 1530-position Partial Deletion matrix, the difference was even larger (2ΔlnL = 316.108; df = 35; *p* = 1.72 × 10^−46^). Branch-to-branch rate heterogeneity is therefore a robust property of *ras85D* 3′ UTR evolution and is not caused by one particular strategy for handling gapped sites.

RelTime parameters were sensitive to alignment filtering. Partial Deletion included 1530 sites, 771 common sites, and 213 invariant sites. Under T92+G+I, γ = 0.97662, I = 0.13922, Ts/Tv = 0.849, lnL = −10,256.936, AICc = 20,664.287, and BIC = 21,280.621. Complete Deletion included 391 sites, 165 constant sites, γ = 0.61121, Ts/Tv = 0.878, lnL = −3163.888, AICc = 6478.569, and BIC = 7046.248. AICc and BIC were not compared directly because the models were fitted to matrices of different sizes and compositions.

The calibrated root age was 44.931 Ma under Partial Deletion and 40.065 Ma under Complete Deletion. Terminal relative-rate distributions were right-skewed, especially for the conservative Complete Deletion core, and were not treated as Gaussian. Median terminal relative rates were similar: 0.597 (IQR 0.342–0.976; range 0.073–5.814) and 0.615 (IQR 0.401–1.197; range 0.077–16.601), respectively. No systematic median shift was detected (paired Wilcoxon *p* = 0.167), while taxon rankings were preserved (Pearson r = 0.854; Spearman ρ = 0.855; *p* < 4 × 10^−11^). Cumulative root-to-tip rates were higher under Partial Deletion for all 36 taxa: medians were 6.103 × 10^−9^ and 4.782 × 10^−9^ substitutions per site per year (*p* = 2.91 × 10^−11^), with high cross-strategy agreement (r = 0.933; ρ = 0.863). This systematic difference reflects matrix composition and total evolutionary path length rather than acceleration toward terminal lineages.

**Figure 1:**
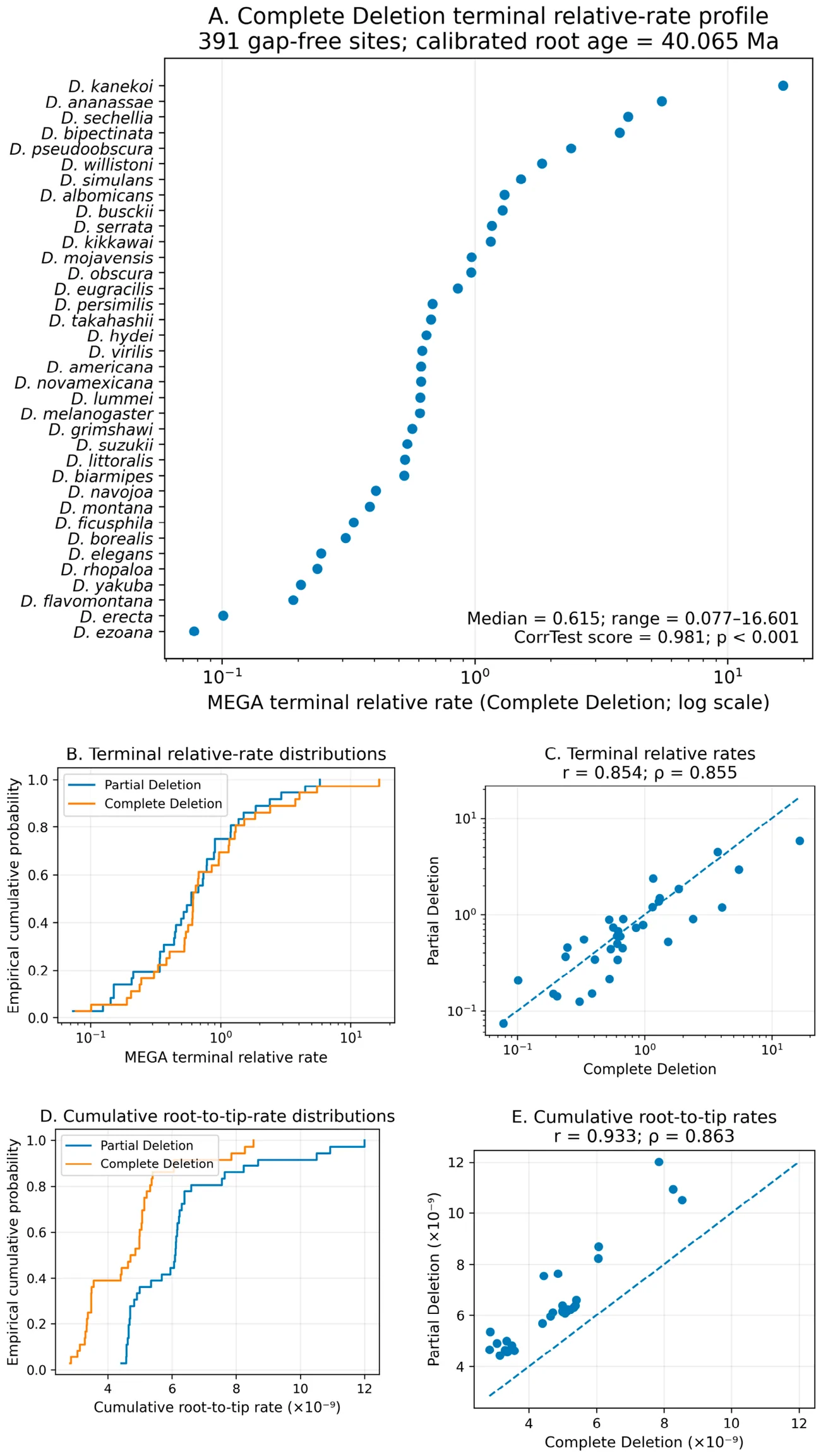
Sensitivity of calibrated RelTime rate estimates to two gap-filtering strategies. (**A**) Terminal relative-rate profile obtained from the Complete Deletion matrix comprising 391 gap-free sites; the calibrated root age was 40.065 Ma. Taxa are ordered by increasing terminal relative rate, and the horizontal axis is logarithmic. The median was 0.615, with a range of 0.077–16.601. CorrTest indicated significant rate autocorrelation (score = 0.981; *p* < 0.001). (**B**) Empirical cumulative distributions of terminal relative rates under Partial Deletion and Complete Deletion. (**C**) Direct comparison of terminal relative rates obtained with the two filtering strategies (Pearson r = 0.854; Spearman ρ = 0.855). (**D**) Empirical cumulative distributions of cumulative root-to-tip rates. (**E**) Direct comparison of cumulative root-to-tip rates (Pearson r = 0.933; Spearman ρ = 0.863). Absolute rates were obtained by calibrating the RelTime tree to the root age shown in Table 2.

CorrTest detected no rate autocorrelation under Partial Deletion (score = 0.0296; *p* > 0.05) but detected strong autocorrelation under Complete Deletion (score = 0.981; *p* < 0.001), demonstrating sensitivity of the autocorrelation inference to site composition (Table 2; Figure 1 and Figure S1).

### 3.2. Microsatellite Variation, Mobile Elements, and Evolutionarily Conserved Motifs

A substantial fraction of small length differences was associated with microsatellite and low-complexity variation. Repeat-number changes were most frequent for (AC)n, (GT)n, and (AT)n arrays and for poly-A and poly-T tracts. In the virilis group, a complex AT-rich segment reached 156 nt in *Drosophila borealis*, contracted to 52 nt in *Drosophila flavomontana*, and persisted as shorter degenerate repeats in other group members, consistent with stepwise expansion and contraction of repeat arrays.

Present-day 3′ UTR length correlated positively with the total number of alignment-derived indel differences (Pearson r = 0.62, *p* < 0.001; Spearman ρ = 0.65). Longer extant sequences therefore contain more traces of insertions and repeat expansions. This does not contradict the deletion bias reconstructed below, because the correlation describes current sequence states whereas ARPIP infers event direction on phylogenetic branches.

Repbase searches identified the three strongest candidate mobile-element traces. In the proximal *Drosophila willistoni* region, a 60 nt fragment approximately 35 nt downstream of the stop codon was 84.5% similar to Gypsy-18_DVir-I. A proximal *Drosophila pseudoobscura* fragment was 89.5% similar to Helitron-2_DSuz and represented an obscura-group event. A large distal *Drosophila grimshawi* interval contained a region 78.3% similar to Gypsy-18_DVir-I. In *D. willistoni*, the marked insertion was unique; in *D. pseudoobscura*, it showed pronounced homology to two other members of the obscura group; and the insertion in *D. grimshawi* was present, in varying degrees of degeneration, in all representatives of the subgenus *Drosophila*. These cases show that mobile elements contributed to some large insertions but were far less frequent than the total indel count (Table 3).

MEME identified 17 statistically independent ECM models, each 29–30 nt long. The number of primary sites per motif ranged from 24 to 37, and motif-model E-values ranged from 7.3 × 10^−399^ to 9.9 × 10^−132^. MAST detected combined motif signatures in every sequence in the original dataset; combined *p*-values ranged from 3.1 × 10^−188^ to 2.05 × 10^−29^. These values describe the fit of the complete motif set and not the functional significance of individual matches.

The comparative map showed that the relative order of ECMs was more conserved than the lengths of the intervals separating them. The most similar compact configurations occurred in the melanogaster, obscura, and virilis lineages, whereas *D. willistoni*, *S. lebanonensis*, *Drosophila busckii*, *Drosophila albomicans*, and *D. grimshawi* displayed wider intervening regions, additional matches, and occasional reverse-complement motif orientations. ECMs therefore form an independent conserved layer of 3′ UTR architecture in which motif repertoire and approximate order are retained despite substantial rearrangement of spacing and orientation.

**Figure 2:**
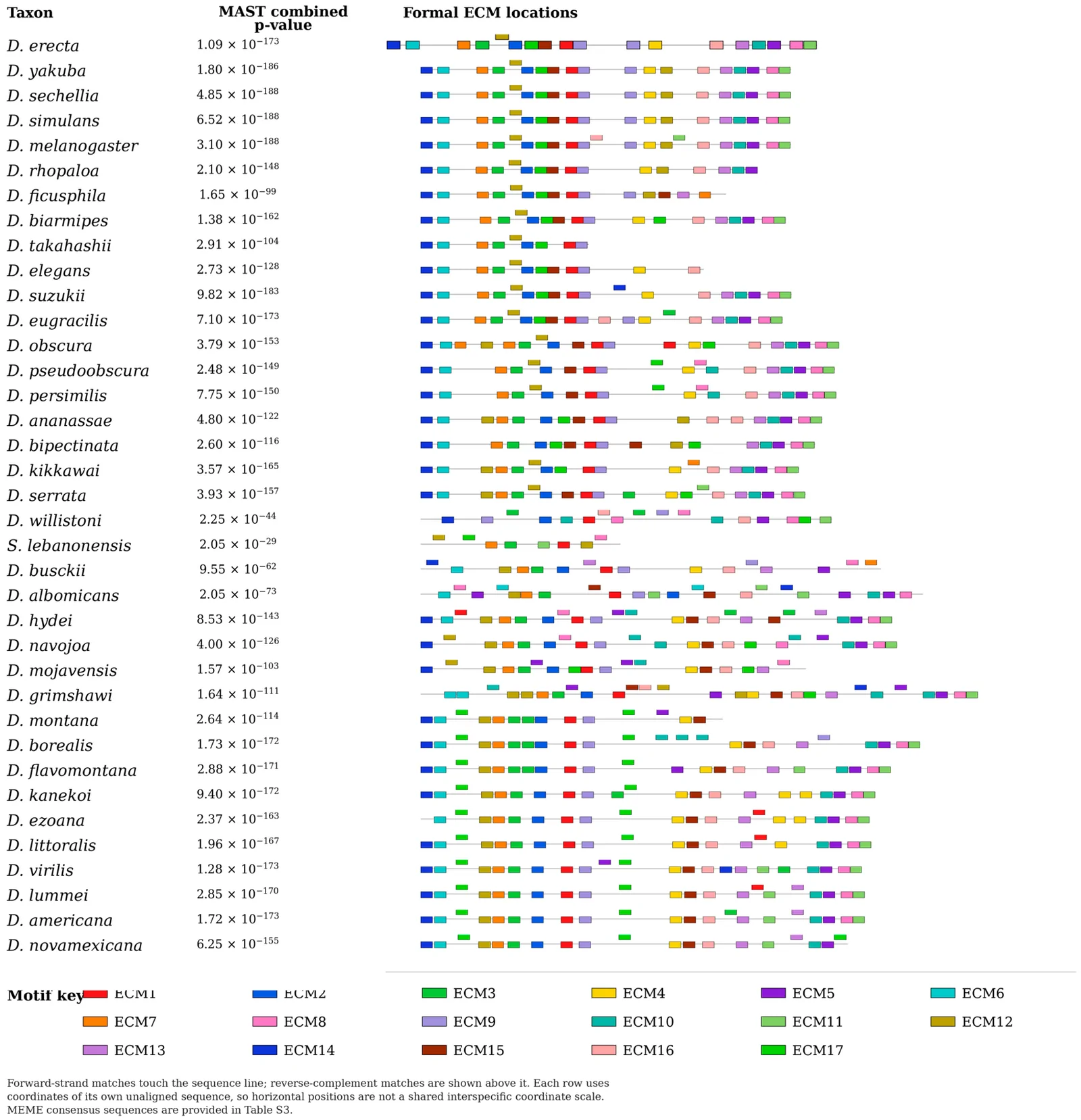
Comparative distribution of 17 evolutionarily conserved motifs in the *ras85D* 3′ UTR based on MAST. All 37 taxa of the final dataset are shown. For each taxon, the MAST combined *p*-value and formal locations of matches to the 17 color-coded ECMs are presented. Blocks touching the horizontal sequence line indicate forward-strand matches; blocks positioned above and not touching the line indicate reverse-complement matches. Each row uses coordinates of its own unaligned sequence, and line length is proportional to the corresponding sequence length. Consequently, horizontal motif positions do not constitute a shared interspecific coordinate scale and should not be interpreted as direct nucleotide homology among species. The color key identifies ECM1–ECM17; MEME consensus sequences and motif parameters are provided in Table S3 and Data S3. All coordinates represented in this figure are species-specific coordinates of the corresponding unaligned 3′ UTR sequence.

Because each row in the MAST map is drawn in its own unaligned sequence coordinates, horizontal motif positions cannot be interpreted as direct nucleotide homology among species. Cross-species positional comparisons and all subsequent ECM analyses used the curated multiple-alignment coordinate system and the final ECM occurrence catalogue (Figure 2; Supplementary Methods SM5; Table S3; Data S3).

### 3.3. Phylogenetic Reconstruction of Indels and Definition of IELs

ARPIP reconstructed 959 block events across the tree: 249 insertions and 710 deletions. Among the 63 positive-length ingroup branches used for branch-level inference, 16 were deletion-enriched, three were insertion-enriched, one showed bidirectional turnover, and 43 showed no significant enrichment after FDR correction.

**Figure 3:**
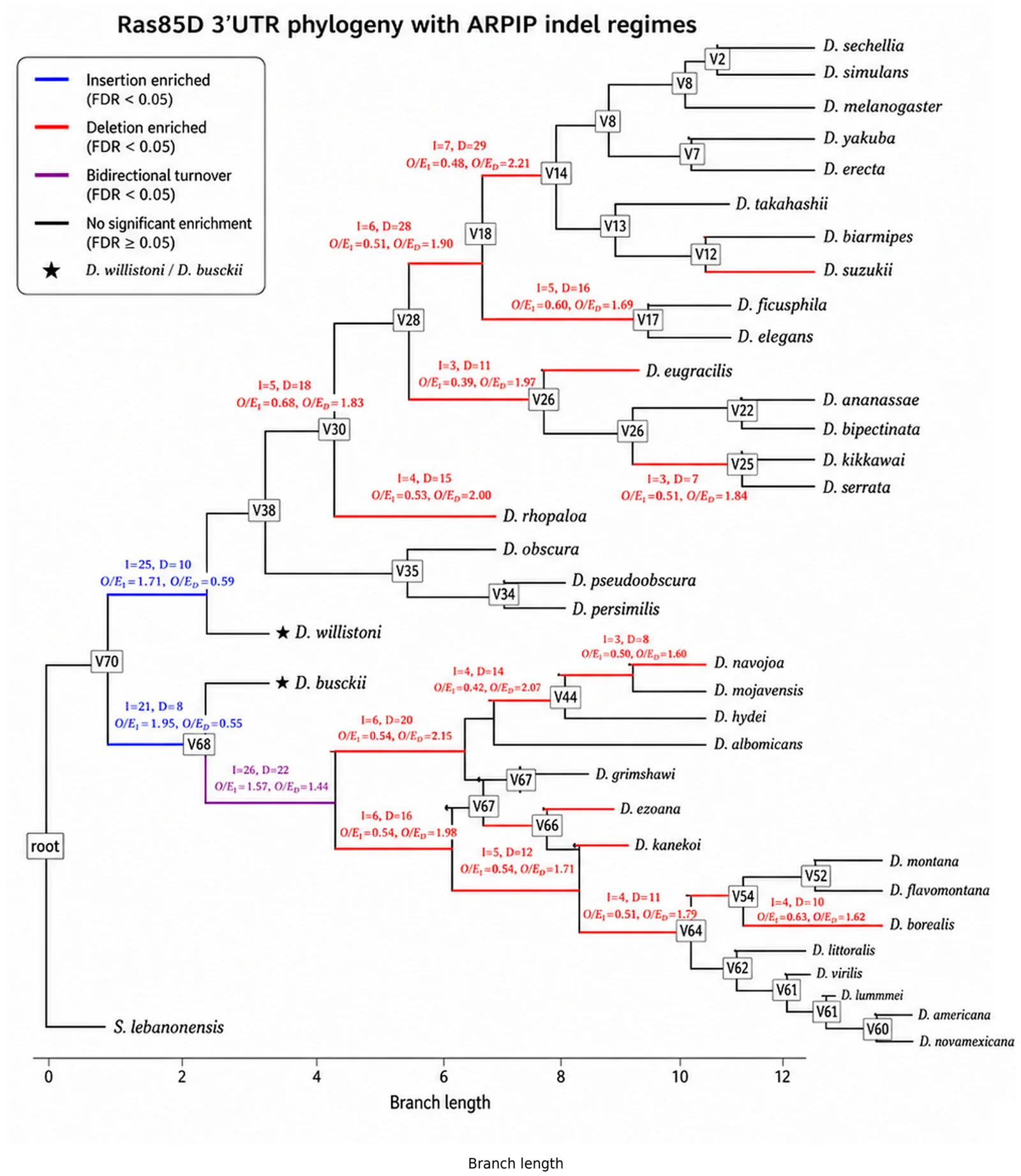
*Ras85D* phylogeny and branch-specific insertion–deletion regimes inferred with ARPIP. Branch color denotes FDR-significant enrichment: blue, insertions; red, deletions; purple, bidirectional turnover; black, no significant enrichment. Selected branches are annotated with insertion (I) and deletion (D) counts and observed-to-expected ratios. *D. willistoni* and *D. busckii* are marked with stars. Historical internal-node labels (Vxx) are retained on the tree for correspondence with the original analytical output; their mapping to the stable N01–N34 branch identifiers is provided in Table S4 and Data S2. *S. lebanonensis* is the outgroup. The horizontal axis represents branch length in the canonical phylogeny used for the ARPIP analysis.

The strongest insertion enrichment occurred on internal branches leading to *D. willistoni* and *D. busckii* and on selected terminal or internal lineages; deletion enrichment was distributed more broadly across the tree. The branch leading to the common lineage represented by V68 showed bidirectional turnover. The final tree, branch regimes, and correspondence between historical Vxx labels and stable N01–N34 identifiers are shown in Figure 3 and Table S4.

Negative-binomial regression with branch length as the exposure showed a marked decline in event counts with increasing normalized root distance. For insertions, β = −4.156, rate ratio = 0.0157, FDR = 2.55 × 10^−7^; for deletions, β = −2.286, rate ratio = 0.102, FDR = 0.0406. After root distance was included, the internal-versus-terminal indicator was not significant.

The *D. willistoni* terminal branch contained 41 events (31 deletions and 10 insertions); mean deletion length was 11.0 nt versus 4.78 nt in other terminal branches (FDR = 0.0136). The *D. busckii* branch contained 49 events (40 deletions and nine insertions), without a significant difference in mean event length after correction.

Spatial aggregation of recurrent events yielded 34 IELs occupying 123 of 2247 alignment columns (5.47%). Projection onto *D. melanogaster* produced 27 unique reference loci. IEL positions were non-random: the coefficient of variation of inter-locus spacing and the maximum spacing both differed from the null distribution (*p* = 0.00698 and *p* = 0.01079, respectively).

### 3.4. Mosaic Conservation of ECMs and Functional Elements

The final functional annotation for *D. melanogaster*, *D. yakuba*, and *D. virilis* comprised 390 primary objects, including 49 ECM occurrences, APA-associated elements, and miRNA targets. ECMs were conserved in a mosaic fashion: some motifs retained clear orthologous positions in all three species, whereas others were absent, displaced, or present in reverse-complement orientation in one lineage.

**Figure 4:**
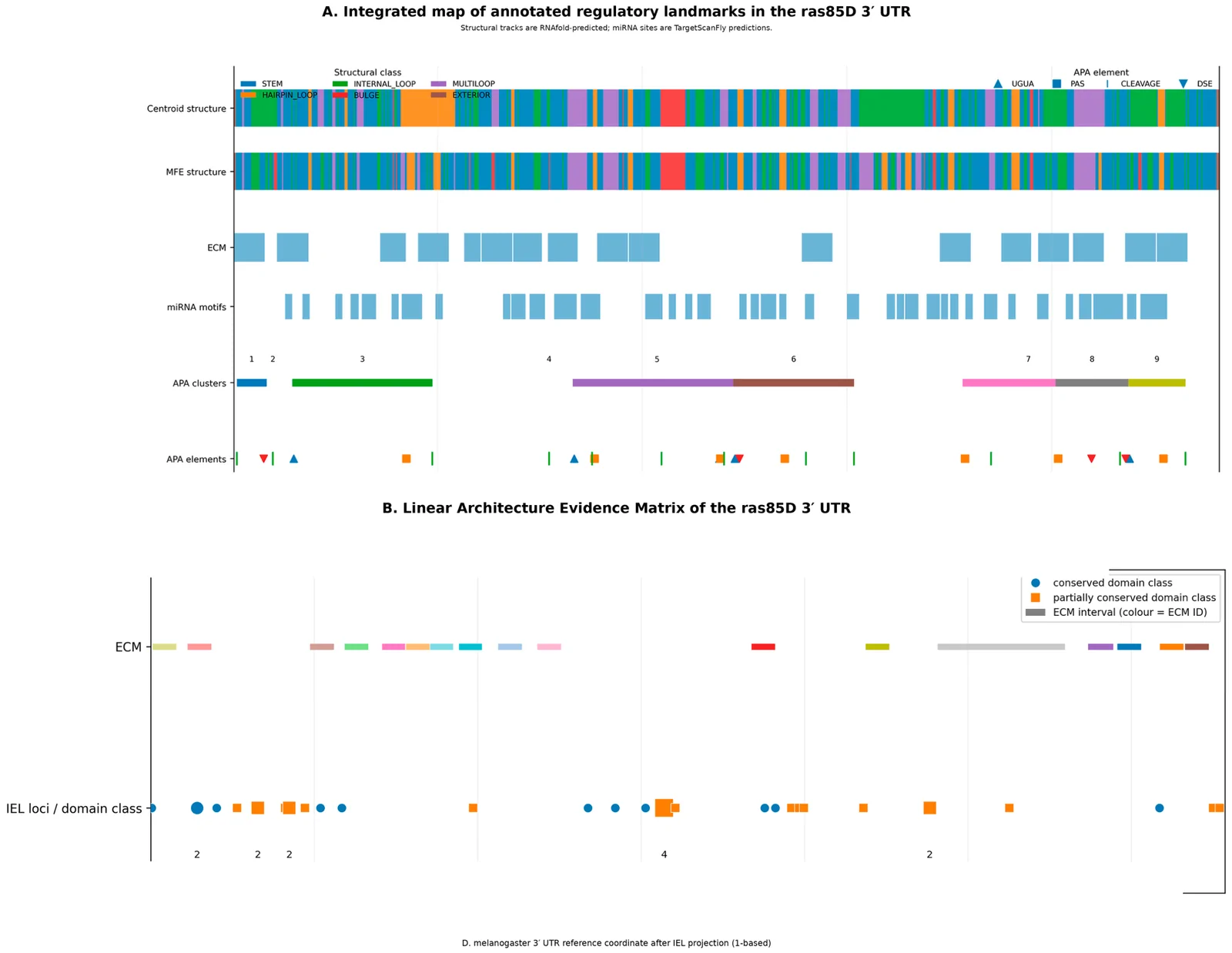
Integrated map of annotated regulatory landmarks and architectural domains in the *ras85D* 3′ UTR. (**A**) Linear *D. melanogaster* map showing nucleotide-level RNAfold-predicted centroid and MFE structural classes, ECM intervals, predicted TargetScanFly miRNA target sites, nine descriptive APA clusters, and individual UGUA, PAS, cleavage-site, and DSE components. (**B**) Linear Architecture Evidence Matrix: ECM intervals are compared with 27 *D. melanogaster* loci onto which 34 IELs project. Marker shape indicates the interspecific consensus architectural class (conserved or partially conserved), and marker area indicates the number of IELs projected to the same locus. Structural-class colors in panel (**A**) denote the six RNAfold classes; APA-cluster colors distinguish C001–C009. In panel (**B**), ECM interval colors identify individual ECMs. These colors are categorical and do not encode quantitative values. Panel A uses 1-based *D. melanogaster* 3′ UTR coordinates (1–964); panel (**B**) uses the same *D. melanogaster* reference coordinate system after projection of IELs from the curated multiple alignment. Structural and miRNA layers shown here are computational predictions; their overlap or proximity to other annotation layers should not be interpreted as evidence of direct physical or molecular interaction.

The integrated *D. melanogaster* map showed that ECMs, miRNA targets, APA clusters, and structural segments are interleaved rather than organized into strictly separated blocks. The 27 reference loci containing projected IELs were distributed across the complete 3′ UTR and frequently occurred at or near ECM boundaries (Figure 4).

Conserved miRNA targets were embedded within both conserved and remodeled segments. Their retention did not require complete conservation of neighboring motifs or a fixed local structural class. The mosaic pattern therefore reflects selective preservation of individual functional landmarks within a sequence that undergoes substantial turnover of intervening regions.

### 3.5. Evolutionary Turnover of Boundary Interfaces

The three strict species graphs contained 50, 39, and 20 functional boundary interfaces in *D. melanogaster*, *D. yakuba*, and *D. virilis*, respectively. Grouping by homologous endpoint families yielded 78 evolutionary interface families: 30 were shared by at least two species and 48 were species-specific.

Among shared families, one was present in all three species, 23 were shared by *D. melanogaster* and *D. yakuba*, and six were shared by *D. yakuba* and *D. virilis*. Thus, interface presence and immediate partners turned over extensively even when the underlying functional objects were conserved.

**Figure 5:**
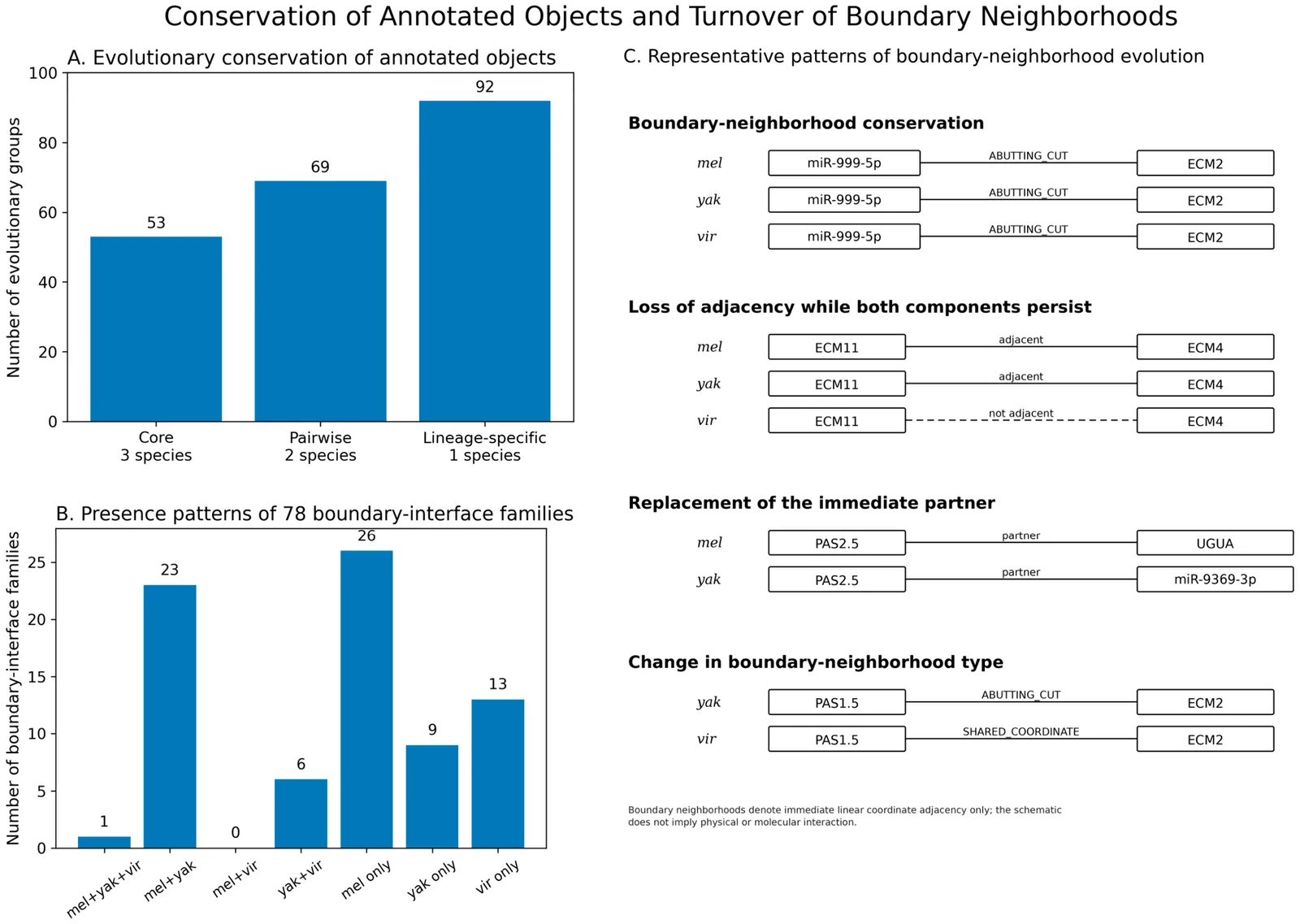
Conservation of annotated objects and evolutionary turnover of boundary neighborhoods. (**A**) Distribution of 214 evolutionary groups of annotated objects among core, pairwise, and lineage-specific categories. (**B**) Interspecific presence patterns of 78 boundary-interface families. (**C**) Representative patterns of boundary-neighborhood evolution: conservation of the miR-999-5p→ECM2 neighborhood across all three focal species; loss of immediate adjacency while both annotated components persist; replacement of the immediate partner; and the single detected change in boundary-neighborhood type for PAS1.5→ECM2 between *D. yakuba* and *D. virilis*. ABUTTING_CUT and SHARED_COORDINATE denote the operational coordinate configurations defined in Methods. Boundary neighborhoods refer exclusively to immediate linear coordinate adjacency and do not imply direct physical or molecular interaction.

Nevertheless, the configuration of an interface was highly stable when the same homologous pair persisted: 29 of 30 multispecies families retained the same boundary-neighborhood type. The only inferred type change involved PAS1.5→ECM2, represented as ABUTTING_CUT in *D. yakuba* and SHARED_COORDINATE in *D. virilis*; the exact branch placement of this change could not be resolved. The main patterns of boundary-interface conservation and turnover are summarized in Table 4 and Figure 5.

### 3.6. RNA Secondary Structure as a Plastic Regulatory Framework

The MFE and centroid models divided each sequence into a dense mosaic of stems, hairpin loops, internal loops, bulges, multiloops, and exterior segments. Structural classes changed rapidly among species even where individual ECMs or APA components remained homologous.

Interval-preserving coverage tests did not identify a universal FDR-significant preference of PAS, UGUA, DSE, cleavage sites, ECMs, or miRNA targets for one local structural class. This negative result concerns local class membership and does not exclude higher-level multilayer organization.

The final Functional Distance analysis likewise found no FDR-significant universal minimum-distance relationship between functional classes and fixed PAS or cleavage-site landmarks. Functional motifs can therefore persist while their immediate structural class changes.

At the same time, the aggregate PAT analysis revealed an architectural association between the APA annotation layer and predicted structural-segment boundaries. This relationship is between annotation layers and does not imply direct physical contact or molecular interaction. Local class specificity and higher-level multilayer architecture are therefore distinct properties.

### 3.7. Coverage Enrichment and Functional Distance

The final *D. melanogaster* dataset contained 510 objects: nine PAS, 12 cleavage sites, 11 UGUA elements, seven DSEs, 90 miRNA sites, 17 ECMs, and 364 structural segments. Coverage Enrichment generated 202 region comparisons and 1414 statistical tests.

Six rows remained significant after FDR correction. Four were geometrically expected positive controls because PAS or cleavage sites were tested within windows centered on the same object class; these rows were not interpreted as independent biological enrichment.

Two independent statistical results involved predicted weakly conserved miRNA target sites. No nucleotide covered by the predicted weak-miRNA layer overlapped ECM_15 (coordinates 1–30) or ECM_11 (230–258), whereas 220 of 459 nucleotides outside these ECMs were covered. Both comparisons had FDR = 0.019548, supporting depletion of predicted weakly conserved miRNA target-site coverage from these two ECMs. Because both layers are computational annotations, this result is an association between annotation layers rather than direct evidence of ECM function or regulation of miRNA binding.

**Figure 6:**
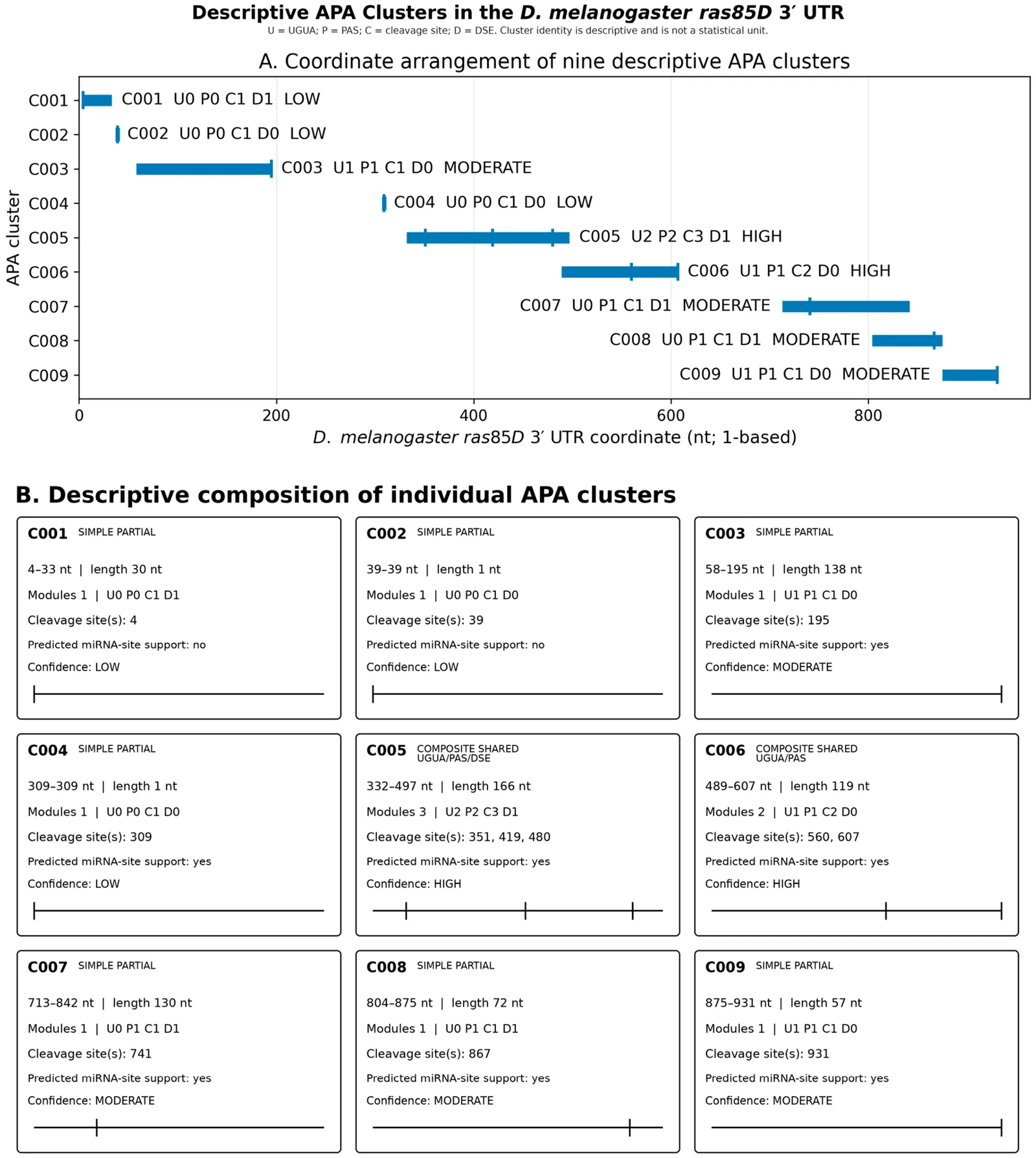
Descriptive organization of nine APA clusters in the *D. melanogaster ras85D* 3′ UTR. (**A**) Coordinate arrangement of clusters C001–C009 and their constituent cleavage sites. U, P, C, and D denote the numbers of UGUA elements, PAS, cleavage sites, and DSEs, respectively. (**B**) Descriptive composition of individual clusters, including coordinates, lengths, component composition, predicted miRNA-site support, and confidence. C005 and C006 are high-confidence composite configurations; C007 and C008 partially overlap but are retained as separate descriptive units. Cluster identity was used only for visualization and was not a statistical unit of the final coverage/distance or PAT analyses. All interval coordinates are 1-based *D. melanogaster* 3′ UTR sequence coordinates.

Functional Distance generated 12 tests to PAS and cleavage sites; none was nominally or FDR-significant. The preliminary tendency for conserved miRNA sites to lie closer to PAS was not supported after adoption of the final six-site conserved annotation (observed median 12 nt; null median 26 nt; *p* = 0.298170; FDR = 0.596340). The descriptive organization of the nine APA clusters is shown in Figure 6.

### 3.8. Structural Separation of APA Components and Between-Context Dispersion

DIFFERENT_MULTILOOP_ARMS did not show significant absolute excess variance relative to its own null model: observed weighted variance was 1.115933 versus a null mean of 1.023529 (ratio 1.090; p_high = 0.161408; FDR = 0.807042). In contrast, SAME_MULTILOOP_INTERVAL showed markedly reduced between-context variance: 0.338645 versus a null mean of 1.228710 (ratio 0.276; two-sided *p* = 0.004660; FDR = 0.023300).

The primary DIFFERENT_MULTILOOP_ARMS − SAME_MULTILOOP_INTERVAL contrast was 0.777288 and was significant under within-species permutation of complete structural profiles (*p* = 0.001490; FDR = 0.004470). The contrast persisted when only head, ovary, and testis were analyzed (difference 0.733902; FDR = 0.027570) and between embryo 0–12 h and embryo 12–24 h (difference 0.294214; FDR = 0.030910).

The direction was positive in all three species and persisted in every leave-one-species-out analysis. It was supported by consensus and centroid annotations, component-only pairs, and pairs involving the cleavage site; the MFE-only analysis retained a positive direction but was not significant. No monotonic relationship was found between the continuous fraction of DIFFERENT_MULTILOOP_ARMS and variance at an individual site, indicating a contrast between architectural regimes rather than a linear dose effect.

**Figure 7:**
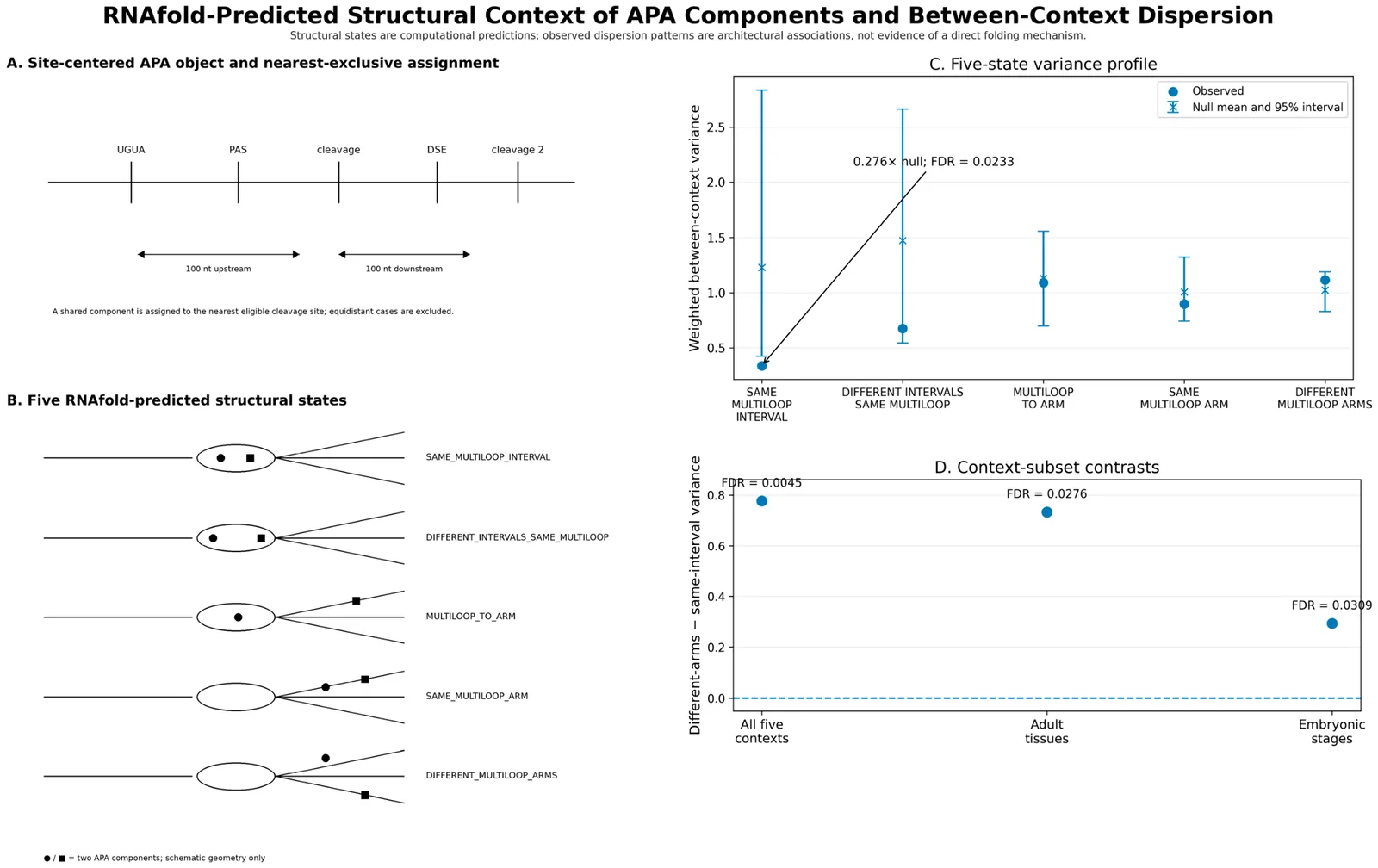
RNAfold-predicted structural context of APA components and between-context dispersion of cleavage-site usage. (**A**) Site-centered APA object and nearest-exclusive component assignment within 100 nt upstream and downstream windows. A component shared by more than one eligible cleavage site was assigned to the nearest site; equidistant cases were excluded. Panel A represents relative distances from the focal cleavage site and does not use an absolute species-specific sequence coordinate system. (**B**) Five mutually exclusive states describing the relative placement of APA-component pairs with respect to RNAfold-predicted multiloop intervals and structural arms. The two symbols represent the paired APA components and are used only to illustrate relative geometry. (**C**) Observed weighted between-context variance for the five predicted structural states compared with the corresponding permutation-null means and 95% intervals. SAME_MULTILOOP_INTERVAL showed FDR-significantly reduced variance relative to its state-specific null (observed/null ratio = 0.276; FDR = 0.0233), whereas absolute hypervariability of DIFFERENT_MULTILOOP_ARMS was not supported. (**D**) Difference in weighted variance between DIFFERENT_MULTILOOP_ARMS and SAME_MULTILOOP_INTERVAL for all five contexts, adult tissues only, and early versus late embryonic stages; all three contrasts were FDR-significant. Structural-state assignments are computational predictions derived from RNAfold. The observed dispersion patterns therefore represent associations between predicted structural context and cleavage-site usage heterogeneity and should not be interpreted as evidence of a direct folding mechanism or molecular interaction. The analysis describes between-context variation rather than biological-replicate variance within a tissue.

The data therefore do not support absolute hypervariability of the different-arm configuration relative to arbitrary structural assignments. Instead, placement of APA components within one multiloop interval is associated with unusually stable site usage, whereas separation between different arms permits a significantly wider range of tissue- and stage-dependent changes (Table S10; Figure 7 and Figure S5).

### 3.9. Pairwise Architecture Test, AEM, and Basal Architecture Network

The final PAT generated 43 unique metrics for three multilayer edges. The only FDR-significant primary association was predicted RNA structure–APA: the observed boundary-to-center ratio was 22.272727 versus a null mean of 4.272117 (O/E = 5.213510; *p* = 0.003802; FDR = 0.007605), indicating preferential placement of APA objects near boundaries in the predicted structural annotation. Because these boundaries were defined from computational RNAfold models, this result represents an architectural association between annotation layers rather than evidence of direct physical contact or molecular interaction.

For ECM–APA, the strongest primary metric was total overlap (O/E = 1.185584; *p* = 0.147529; FDR = 0.216730); for ECM–RNA structure, it was the boundary-to-center ratio (O/E = 3.508784; *p* = 0.155894; FDR = 0.311787). Both edges remained exploratory and lacked FDR-significant primary support.

The AEM was rebuilt for 34 IELs across three species and contained 102 IEL × species rows. All IELs received a resolved interspecific consensus: 14 were classified as conserved domain class and 20 as partially conserved domain class; no species-variable or unresolved classes remained.

In the final BAN, IEL–ECM and predicted RNA structure–APA were FDR-supported. IEL–predicted RNA structure was retained as supported by structural-boundary preference, whereas IEL–APA showed no global effect. ECM–APA and ECM–predicted RNA structure were represented as nominal exploratory relationships without FDR-significant primary associations.

An architectural domain in the AEM denotes the contextual class of a specific IEL defined by the joint ECM, APA, and secondary-structure environment; it is not a separate coordinate interval and is not synonymous with a secondary-structure domain. Full metrics, the AEM, and the evidence network are provided in Table S8, Figure S4, and Data S4. Key results of the Coverage Enrichment, Functional Distance, between-context dispersion, and PAT analyses are summarized in Table 5.

### 3.10. Integrated View of ras85D 3′ UTR Evolution

The results identify several interconnected evolutionary levels. At the primary-sequence level, the locus shows deletion-biased and branch-heterogeneous turnover; mobile elements explain some large insertions, and microsatellites explain local length variation. At the linear-architecture level, ECMs and subsets of miRNA and APA elements persist, whereas intervals and immediate partners change. At the predicted-structure level, no universal assignment of each motif class to one local structural class was found, but the aggregate APA layer was enriched near predicted structural boundaries. At the context-dependent level, placement within one multiloop interval was associated with reduced usage variance, whereas different-arm configurations were associated with a wider tissue- and stage-dependent range. At the integrative level, most IELs retained fully or partly similar interspecific architectural contexts.

The extant *ras85D* 3′ UTR was therefore generated not by conservation of one fixed configuration but by a balance among mutational renewal, preservation of selected regulatory landmarks, and a permissible set of local architectural solutions. The final coverage/distance, dispersion, and PAT/AEM/BAN analyses distinguish local class-specific effects from robust multilayer relationships.

## 4. Discussion

### 4.1. Nucleotide-Substitution Rates Relative to Coding and Putatively Neutral Sequences

The *ras85D* 3′ UTR shows pronounced rate heterogeneity among branches, and this conclusion is robust to gap-filtering strategy. At the same time, numerical rate estimates depend strongly on which alignment columns are retained. Partial Deletion incorporates variable regions and yields longer cumulative root-to-tip paths, whereas Complete Deletion describes a conservative core.

The observed rates fall within the broad range previously reported for *Drosophila nuclear* non-coding sequences, but direct comparison with coding regions or putatively neutral short introns is limited by differences in taxon sampling, calibration, and genomic context [43,44]. Mutation-accumulation experiments demonstrate substantial heterogeneity in spontaneous substitution and indel processes [45,46], while synonymous and short-intron sites may themselves be subject to selection [47,48].

Consequently, we interpret the RelTime results as evidence of relative branch-to-branch heterogeneity within this locus, not as a universal estimate of a neutral substitution rate. Matched coding-sequence and short-intron controls from the same taxa and genomic neighborhoods will be required for a direct quantitative comparison.

### 4.2. Deletion Bias and the Contribution of Insertion–Deletion Variation

The excess of reconstructed deletions over insertions agrees with the well-documented deletion bias of *Drosophila genomes* [49,50]. However, branch-level regimes were heterogeneous: several lineages showed insertion enrichment, and one internal lineage showed bidirectional turnover. Thus, a genome-wide deletion bias does not imply uniform local dynamics.

Present-day sequence length correlated positively with alignment-derived indel differences, whereas ancestral reconstruction showed more deletions. These results describe different aspects of the process. Extant length integrates surviving insertions, repeat expansions, and deletions over the complete history; ARPIP assigns event direction to branches. The two observations are therefore compatible.

Microsatellite expansions and contractions provide a natural mechanism for many short length changes, whereas mobile-element candidates explain only a small subset of large insertions. Similar combinations of repeat turnover and occasional transposable-element activity have been described in rapidly evolving non-coding regions [51,52].

### 4.3. Non-Random Indel Distribution and the Biological Meaning of IELs

IELs summarize regions that are repeatedly involved in indel evolution across different branches. They should not be interpreted as single ancestral events or as fixed functional elements. Their value lies in identifying spatially recurrent remodeling of the alignment.

The non-random spacing of IEL projections and their association with ECMs indicate that sequence turnover is concentrated in a limited set of architectural neighborhoods. This concentration may arise because conserved motifs constrain the positions at which indels can be tolerated, or because repetitive and structurally flexible intervals near motif boundaries are intrinsically more mutable.

The finding is consistent with previous demonstrations that functional non-coding elements can persist despite rapid turnover of the surrounding sequence [53,54,55,56]. The distinctive feature here is the integration of phylogenetically reconstructed indels with motif, APA, miRNA, and structural layers within one 3′ UTR.

### 4.4. Mosaicism and Modularity of Regulatory Architecture

The *ras85D* 3′ UTR is not conserved as a single continuous block. Instead, individual ECMs, miRNA targets, and APA elements are retained within a background of variable intervals. This mosaic pattern resembles turnover documented for other cis-regulatory systems [54,55,56].

Boundary-interface analysis adds a local architectural dimension. Immediate partners change frequently, but when the same homologous pair persists, the boundary-neighborhood configuration is almost always conserved. Selection may therefore act on a limited set of permissible adjacency configurations rather than on complete preservation of all local partners.

The single PAS1.5→ECM2 configuration change shows that even boundary type can evolve, but this appears much rarer than interface gain or loss. Broader taxon sampling and experimental annotation will be needed to determine whether this asymmetry is a general property of 3′ UTR architecture.

### 4.5. Functional and Structural Load of Evolutionarily Conserved Fragments

ECMs form the most persistent linear landmarks in the locus, but their designation denotes evolutionary sequence conservation rather than experimentally demonstrated function. A conserved motif could, depending on context, contribute to RNA-binding-protein recognition, local folding, overlap with a miRNA site or an APA component, or spacing between neighboring elements; these possibilities remain mechanistic hypotheses unless tested experimentally.

The lack of universal enrichment of ECMs, predicted miRNA targets, or individual APA components in one predicted structural class does not imply that secondary structure is irrelevant. Rather, the PAT showed that the aggregate APA layer is preferentially associated with predicted structural-segment boundaries. A regulatory role could therefore be compatible with several predicted local structural classes while a higher-level association between annotation layers remains detectable.

RNA structural plasticity has been documented in other regulatory 3′ UTRs, including *bicoid* mRNA [57]. Combinatorial action of RNA-binding proteins, non-coding RNAs, and RNA granule components can read such alternative structural contexts [58,59,60].

### 4.6. Functional Architecture: Coverage, Distances, and Multilayer Relationships

Comparative studies show that 3′ UTR function depends on sequence, position, and joint context of regulatory elements. Our coverage results do not support a simple model in which every motif class is enriched in one structural state, but they identify two local exceptions: ECM_15 and ECM_11 are depleted of predicted weakly conserved miRNA target-site coverage. These patterns are consistent with localized constraint in selected conserved intervals, but the computational annotations do not identify the underlying mechanism.

The absence of significant Functional Distance effects indicates that proximity to PAS or a cleavage site is not a universal property of the annotated classes. The preliminary conserved-miRNA→PAS tendency disappeared after adoption of the final annotation, emphasizing that the object set must be fixed before distance results are interpreted.

The strongest positive signal was multilayer rather than a single distance: predicted RNA structure–APA was the only FDR-significant primary PAT edge. Preferential placement of APA objects near predicted structural boundaries is compatible with the hypothesis that processing may be related to transitions between local structural segments. However, because the structural segments are computational predictions, the result should be interpreted as an architectural association between annotation layers, not as evidence that APA factors physically recognize such boundaries or that a particular predicted fold exists in vivo.

The AEM adds an interspecific perspective: all 34 IELs had resolved consensus classes; 14 retained the same architectural class across all three species and 20 retained it partially. Recurrently remodeled regions are therefore not architecturally disorganized; they often preserve a characteristic combination of ECM, APA, and structural context despite primary-sequence change.

Experimental tests will require SHAPE or DMS structure probing, independent 3′-end mapping, reporter constructs that alter structural boundaries and local ECM/miRNA context, and binding data for cleavage and polyadenylation components.

### 4.7. Structural Separation of APA Components and Regulatory Plasticity

The initial mean-effect test did not retain a consistent reduction in cleavage-site usage for APA components located on different multiloop arms after global FDR correction. The dispersion analysis addresses a different biological prediction: structural separation may not impose one directional change but may expand the range of tissue- and stage-dependent regulatory states.

The result supports this model in a relative, not absolute, sense. DIFFERENT_MULTILOOP_ARMS was not hypervariable relative to its own null distribution. The principal signal arose because SAME_MULTILOOP_INTERVAL was unusually stable, making the different-arms versus one-interval contrast robust. Because these state assignments are derived from RNAfold predictions, co-location within one predicted multiloop interval may mark a more integrated regulatory context, whereas assignment to different predicted arms may mark contexts in which RNA-binding proteins, cleavage and polyadenylation factors, miRNAs, and other regulators act more independently.

Context-dependent interpretation of the same cis-architecture may depend not only on the presence of RNA-binding proteins and miRNAs but also on the functional state of the cleavage and polyadenylation machinery. Mitschka and Mayr discuss phosphorylation and ubiquitination of polyadenylation factors as mechanisms capable of altering their stability, localization, interactions, and activity [3]. If the predicted structural arrangement reflects relevant in vivo organization, the same APA module could therefore be processed differently in tissues and developmental stages with different signaling pathways and post-translational-modification profiles. Assignment of components to different predicted multiloop arms need not cause a constant reduction in site efficiency but could be associated with greater dependence of site choice on trans-acting factors.

Post-transcriptional regulation of already formed 3′ isoforms creates an additional layer. An estimated 10–30% of genes with multiple 3′ UTRs may be subject to this type of regulation [3]. Proposed mechanisms include sequential polyadenylation, nuclear RNA degradation, regulation of mRNA export, and control of mRNA stability. The last mechanism is especially relevant here: the observed isoform fraction may reflect both primary cleavage-site choice and subsequent context-dependent stabilization or elimination of the transcript. Over evolutionary time, this may favor not one fixed configuration but a set of 3′ UTR structural states responsive to tissue-specific regulatory systems.

This explanation remains a mechanistic hypothesis. It requires biological replicates, binding profiles for specific proteins, experimental structure mapping, and reporter constructs in which the relative position of APA components is changed without altering their primary sequences. Nevertheless, the consistency of the contrast across tissues, embryonic stages, and leave-one-species-out analyses supports the interpretation of between-context regulatory plasticity.

### 4.8. Study Limitations

The integrated functional–structural model is based on one gene, and interspecific functional-object comparisons are limited to three species. The absence of significance in individual coverage or distance tests therefore does not demonstrate absence of biological effects in other genes or tissues. The tissue-resolved dataset contains one aggregated value per species × tissue/stage combination, so the dispersion analysis characterizes differences among contexts rather than biological-replicate variance within a tissue.

Secondary structures are computational predictions rather than experimentally measured conformations. Centroid and MFE models represent different views of the structural ensemble; consequently, the association of APA objects with predicted segment boundaries requires independent experimental validation and should not be interpreted as direct three-dimensional contact, molecular interaction, or proof that the modeled fold is adopted in vivo. Likewise, conservation or statistical association of computationally identified ECMs and predicted miRNA sites does not by itself demonstrate biological function.

Phylogenetic indel reconstruction depends on alignment, topology, and the Poisson Indel Process model. IELs reduce dependence on any single event assignment but do not remove coordinate uncertainty in highly gapped regions. Rate comparisons with coding and putatively neutral sequences are also context-dependent because matched CDS and short-intron controls from the same genomic neighborhood were not analyzed.

Finally, tests were organized into predefined FDR families, but architectural features are not independent. Four FDR-significant coverage rows were internal positive controls for APA-centered windows and were not treated as independent biological evidence. Larger datasets should be analyzed with hierarchical models and external replication.

### 4.9. An Integrated Model of ras85D 3′ UTR Evolution

The study-specific terms used here refer to successive analytical levels rather than equivalent biological entities. IELs summarize recurrently remodeled regions identified from phylogenetically reconstructed indels, whereas ECMs are conserved sequence motifs identified independently. The PAT tests pairwise spatial relationships among ECM, APA, and predicted RNA-structure layers. The AEM then summarizes the combined ECM, APA, and structural context of each IEL across the three focal species, and the BAN provides a final evidence network that records which relationships are FDR-supported, otherwise supported, exploratory, or unsupported.

The data support a five-level model. The first level is mutational flux: point substitutions, deletion-biased indel processes, microsatellite changes, and rare large mobile insertions. The second consists of spatially restricted IELs. The third is conservation of subsets of ECM, miRNA, and APA elements despite turnover of intervals and immediate partners. The fourth is context-dependent regulatory plasticity of APA components: co-location within one predicted multiloop interval is associated with reduced variance, whereas assignment to different predicted arms is associated with a wider range of states. The fifth is multilayer architecture, in which IEL–ECM and RNA structure–APA have FDR-supported evidence and most IELs retain fully or partly similar interspecific architectural classes.

The target of selection need not be the exact primary sequence or a single structure. Selection may maintain a functional repertoire and a range of permissible architectural states: retention of a key motif, an acceptable boundary neighborhood, APA position relative to structural boundaries, and a stable composition of the architectural domain.

The final BAN distinguishes FDR-supported, supported, nominal, and negative relationships, thereby separating architectural integration from physical interaction. The complete evidence network and the origin of each edge are provided in Figure S4 and Table S8.

## 5. Conclusions

The *ras85D* 3′ UTR evolves as a mosaic regulatory system. Branch-to-branch substitution-rate heterogeneity is reproduced under different filtering strategies, and indel evolution is characterized by deletion bias and recurrent involvement of a limited set of regions. Rare mobile insertions and microsatellites contribute to, but do not exhaust, this process.

Evolutionary conservation is expressed primarily as retention of selected regulatory landmarks and their relative order rather than invariance of the entire local configuration. Boundary-interface presence and immediate partners turn over extensively, while the configuration of a persistent neighborhood is almost always retained. Predicted RNA secondary-structure models provide a plastic architectural framework in which similar annotated regulatory elements can occur in multiple topological contexts.

Local architecture is not a simple sum of PAS, UGUA, DSE, or miRNA sites. Weakly conserved miRNA sites are depleted only in selected ECMs, and no universal minimum-distance effect to PAS or cleavage sites was found. The aggregate APA layer is nevertheless associated with structural-segment boundaries, and all IELs retain fully or partly similar interspecific architectural contexts. Placement of APA components within one predicted multiloop interval is associated with reduced between-context variance, whereas assignment to different predicted arms is associated with a wider tissue- and stage-dependent range. Overall, *ras85D* 3′ UTR evolution reflects a balance among mutational renewal, preservation of conserved and experimentally anchored regulatory landmarks, and plasticity of multilayer architecture.

## Figures and Tables

**Table 1:** Hierarchy of datasets and statistical models used in this study.

Analysis Level	Dataset	Primary Unit	Key Null Model/Model
Nucleotide substitutions	37 taxa	branch/alignment site	T92+G; T92+G+I; RelTime
Indels	37 taxa	block event/branch	ARPIP; O/E; negative-binomial regression
IEL–ECM	34 IELs; 27 *D. melanogaster* loci	interval	interval permutation; circular shift
Annotated objects	*D. melanogaster*, *D. yakuba*, *D. virilis*	object/orthology group	aligned coordinates and object type
Boundary interfaces	three strict adjacency graphs	directed adjacent pair	conditional randomization within BPU
Coverage/distance	510 *D. melanogaster* objects	object/region/landmark	length-preserving interval randomization; edge-to-edge distance
PAT/AEM/BAN	49 ECMs; 127 APA objects; 1135 RNAfold-predicted structural segments; 34 IELs × 3 species	edge/architectural domain	1314 circular shifts; evidence matrix
Between-context dispersion	27 sites × 5 tissue/developmental contexts	site × context/predicted structural state	site centering; within-species profile permutation

**Table 2:** Comparison of repeated calibrated RelTime analyses under two gap-filtering strategies.

Metric	Partial Deletion, 10% Threshold	Complete Deletion
Number of positions	1530	391
Common positions	771	391
Constant/invariant sites	213; I = 0.13922	165; I = n/a
Gamma	0.97662	0.61121
Ts/Tv	0.849	0.878
lnL	−10,256.936	−3163.888
AICc/BIC	20,664.287/21,280.621	6478.569/7046.248
Root age, Ma	44.931	40.065
Terminal relative rate, median (IQR)	0.597 (0.342–0.976)	0.615 (0.401–1.197)
Terminal relative-rate range	0.073–5.814	0.077–16.601
Cumulative root-to-tip rate, median ×10^−9^	6.103 (4.705–6.389)	4.782 (3.462–5.158)
CorrTest	score = 0.0296; *p* > 0.05	score = 0.981; *p* < 0.001

Note: AICc and BIC characterize the individual analyses and are not used for direct comparison of matrices with different sizes and site compositions.

**Table 3:** Candidate regions homologous to mobile elements. Coordinates are species-specific 3′ UTR sequence coordinates.

Taxon/Lineage	Candidate Element	Class	Homologous Coordinates, nt	Similarity	Alignment Score	Position Relative to ECMs
*D. willistoni*	Gypsy-18_DVir-I	LTR/Gypsy	35–94	84.48%	232	ECM13 region
*D. grimshawi*	Gypsy-18_DVir-I	LTR/Gypsy	1100–1170	78.26%	229	between ECM11 and ECM13
*D. pseudoobscura*/obscura group	Helitron-2_DSuz	DNA/Helitron	40–75	89.47%	224	ECM8 region

**Table 4:** Main characteristics of evolutionary conservation of boundary interfaces.

Metric	Value	Interpretation
Strict species interfaces	50/39/20	*D. melanogaster*/*D. yakuba*/*D. virilis*
Evolutionary interface families	78	unique pairs of homologous groups
Multispecies families	30	1 core; 23 MEL–YAK; 6 YAK–VIR
Same boundary-neighborhood type	29 of 30 (96.7%)	type retained when the pair persists
Boundary-neighborhood type change	1	PAS1.5→ECM2
Species-specific families	48	high turnover of interface presence

**Table 5:** Key results from Coverage Enrichment, Functional Distance, between-context dispersion, and the Pairwise Architecture Test. Region coordinates, where shown, are 1-based *D. melanogaster* 3′ UTR sequence coordinates.

Analysis/Edge	Region/Metric	Effect	p; FDR	Interpretation
Coverage predicted MIRNA_WEAK	ECM_15 (1–30)	0/30 vs. 220/459; RR = 0	0.002300; 0.019548	FDR-supported depletion of predicted sites
Coverage predicted MIRNA_WEAK	ECM_11 (230–258)	0/29 vs. 220/459; RR = 0	0.002100; 0.019548	FDR-supported depletion of predicted sites
Functional Distance	PAS/cleavage; 12 tests	0 significant tests	—	effect not supported
Predicted RNA structure–APA	boundary/center	O/E = 5.213510	0.003802; 0.007605	FDR-supported architectural enrichment
ECM–APA	overlap	O/E = 1.185584	0.147529; 0.216730	not significant
ECM–predicted RNA structure	boundary/center	O/E = 3.508784	0.155894; 0.311787	not significant
Dispersion	one interval/null	V ratio = 0.276	0.004660; 0.023300	reduced variance
Dispersion	different predicted arms—one predicted interval	ΔV = 0.7773	0.001490; 0.004470	broader between-context range

## Data Availability

The data, custom computational scripts, final analysis inputs, machine-readable outputs, and quality-control records supporting the analyses reported in this study are provided in the Supplementary Materials and in the public GitHub repository https://github.com/alexgenego/ras85d-3utr-architecture (accessed on 26 August 2026). The manuscript-associated release (v1.0.0) is permanently archived in Zenodo at https://doi.org/10.5281/zenodo.22108845. The repository includes, in particular, the conditional-randomization procedures used for boundary-neighborhood analyses and the interval-preserving Coverage Enrichment tests, together with PAT/AEM, Functional Distance, and between-context dispersion workflows. Two normalized historical input files used in the original Boundary Pair Universe analysis were not retained; the original analysis scripts, recorded input checksums, QC records, and compact final outputs are preserved, and this limitation is documented explicitly in the repository. Files labeled reconstructed are documented reconstructed datasets derived from preserved final evidence and are not presented as the lost historical originals.

## Supplementary Materials

The following supporting information can be downloaded at https://www.mdpi.com/article/10.3390/genes17091041/s1, Supplementary Methods SM1–SM10; Supplementary Results SR1–SR7; Table S1: Sequence provenance, coordinates and alignment QC; Table S2: RelTime matrices, model parameters, and sensitivity of rate estimates; Table S3: Mobile-element candidates, microsatellite evidence and final ECM catalogue; Table S4: Canonical branch universe and branch-level indel evidence; Table S5: IEL passport, *D. melanogaster* projection and spatial tests; Table S6: Three-species functional-object registry and IEL integration layer; Table S7: Boundary Pair Universe and conservation of boundary-neighbourhood types; Table S8: Final Pairwise Architecture Test, AEM and BAN synthesis; Table S9: Final Coverage Enrichment and Functional Distance analyses; Table S10: Between-context dispersion of cleavage-site usage in five APA-component structural states; Figure S1: Sensitivity of RelTime estimates to alignment-site filtering. Panel A presents the Complete Deletion terminal relative-rate profile; panels B–E compare Partial and Complete Deletion terminal and cumulative root-to-tip rates; Figure S2: Canonical phylogeny with stable internal IDs N01–N34 and final branch-specific indel regimes. The outgroup and zero-length branches are retained for topology but excluded from the 63-branch statistical universe; Figure S3: Distribution of 34 IELs across the 2247-column analytical alignment and their projection to 27 unique *D. melanogaster* loci. Symbols indicate projection class; Figure S4: Four-node evidence network integrating IEL, ECM, RNA secondary structure, and APA. Edge grades preserve the strength and provenance of the underlying tests and do not imply direct three-dimensional molecular contact; Figure S5: Between-context dispersion of cleavage-site usage in five APA-component structural states. (A) Observed weighted variances and 95% intervals of null distributions. (B) DIFFERENT_MULTILOOP_ARMS − SAME_MULTILOOP_INTERVAL contrasts for all five contexts, adult tissues, and embryonic stages. (C) Species-specific direction of the contrast; Data S1: Alignment, coordinates, and sources; Data S2: ARPIP branches and IELs; Data S3: Functional registry and boundaries; Data S4: Final statistical outputs; Data S5: Multiloop context dispersion; Analysis scripts and reproducibility code are available in the public GitHub repository https://github.com/alexgenego/ras85d-3utr-architecture (accessed on 26 August 2026), with the manuscript-associated release (v1.0.0) permanently archived in Zenodo at https://doi.org/10.5281/zenodo.22108845; and the Supplementary Master Index and QC. References cited in the Supplementary Materials are included in the main reference list.
