# Supplementary material for: The Evolutionary Plasticity, Conservation of Functional Motifs, and Structural—Functional Architecture of the ras85D 3′ UTR in Drosophila
Source: Genes (Basel). 2026 Aug 29;17(9):1041. doi: 10.3390/genes17091041 (PMC13606288; doi:10.3390/genes17091041)
Supplement: Supplementary file 1 [file genes-17-01041-s001.zip › 00_Index_and_QC/Data_and_Code_Availability_Statement_EN.pdf]

Data and Code Availability Statement

The compact Supplementary package contains the data, tables, figures, and documentation required to understand and reproduce the analyses reported here. Analysis scripts and reproducibility code are publicly available at https://github.com/alexgenego/ras85d-3utr-architecture, and the fixed manuscript-associated release (v1.0.0) is permanently archived in Zenodo at https://doi.org/10.5281/zenodo.22108845. Extended diagnostic tables, full execution logs, intermediate and legacy analysis versions, additional sensitivity results, and large machine-readable evidence matrices are retained in the internal project archive and are available from the corresponding author upon reasonable request.
