# Supplementary material for: The Evolutionary Plasticity, Conservation of Functional Motifs, and Structural—Functional Architecture of the ras85D 3′ UTR in Drosophila
Source: Genes (Basel). 2026 Aug 29;17(9):1041. doi: 10.3390/genes17091041 (PMC13606288; doi:10.3390/genes17091041)
Supplement: Supplementary file 1 [file genes-17-01041-s001.zip › 01_Methods/Supplementary_Methods_SM1-SM10_EN.pdf]

Supplementary Methods

Evolutionary Plasticity and Architecture of the *ras85D* 3′ UTR in *Drosophila*

Scope, Correspondence, and Quality Control

The final Supplementary package contains only the materials required to verify the principal conclusions and reproduce the key analyses. It comprises Supplementary Methods SM1–SM10, Supplementary Results SR1–SR7, Tables S1–S10, Figures S1–S5, Data S1–S5, and the Supplementary Master Index. Analysis scripts and reproducibility code are maintained in the public GitHub repository https://github.com/alexgenego/ras85d-3utr-architecture and in the manuscript-associated Zenodo release v1.0.0 (https://doi.org/10.5281/zenodo.22108845). Large intermediate matrices, detailed execution logs, replaced versions, and private sensitivity tables are retained in the internal project archive and are available from the corresponding author upon reasonable request.

All coordinate intervals are reported as 1-based inclusive. Taxon names, internal-node identifiers, coordinates, and object statuses were synchronized across files. Only records with final agreed status were used in the reported analyses; legacy and non-final rows were excluded. The final package is tracked by SHA-256 checksums in the file manifest, and the Supplementary Master Index records file presence, section correspondence, and consolidated QC status.

SM1. Sequences, Sources, Alignment, and Coordinates

Thirty-seven drosophilid taxa were analyzed, with *Scaptodrosophila lebanonensis* as the outgroup. Assembly sources, scaffolds, coordinates, orientation, and mapping evidence are provided in Table S1. Ungapped sequences were mapped to the stated assemblies: 36 sequences mapped to one scaffold, whereas *D. borealis* was reconstructed from two scaffolds.

The original alignment contained 2276 columns. The analytical matrix comprised columns 1–2247; terminal columns 2248–2276 were excluded before gap removal. Five technical taxon labels were corrected without changing sequences. Complete alignments, coordinate maps, the tree, QC files, and checksums are included in Data S1.

SM2. RelTime and Sensitivity to Site Filtering

Maximum-likelihood and RelTime analyses were performed in MEGA 12.1.2 using one fixed topology. Partial Deletion with a 10% site-coverage threshold (1530 sites) was compared with Complete Deletion (391 gap-free sites). T92+G+I, five gamma categories, and the same seven calibrations were used in both analyses.

The strict clock was evaluated with a likelihood-ratio test. MEGA relative rates were extracted for 36 terminal ingroup taxa, and cumulative root-to-tip rates were calculated. Vectors were compared using the paired Wilcoxon test and Pearson and Spearman coefficients. Model parameters and taxon-specific values are in Table S2, graphical sensitivity analysis in Figure S1, and reproducibility code in the public GitHub/Zenodo repository cited above.

SM3. ARPIP, Block Events, and Branch Regimes

Insertions and deletions were reconstructed with bpp-ARPIP from the 2247-column analytical alignment and fixed tree. Adjacent coordinates of the same type assigned to the same branch were merged into block events; the final catalogue contained 959 events. Branch statistics were calculated for real positive-

length ingroup branches; zero-length branches, the outgroup, and the synthetic ingroup stem were excluded.

Expected event counts were proportional to the total branch-length fraction. Enrichment was evaluated with upper-tail binomial tests and separate BH correction for insertions and deletions. Branches received stable N01–N34 IDs together with legacy V aliases. The canonical registry and final regimes are in Table S4 and Figure S2; minimal data are in Data S2, and reproducibility code is in the public GitHub/Zenodo repository cited above.

SM4. IEL Definition, Projection, and Spatial Organization

IELs were defined as regions of the analytical alignment containing recurrent or overlapping locations of independently reconstructed indel events. Thirty-four IELs were retained. Projection to *D. melanogaster* used a column-wise coordinate map; several IELs could correspond to one reference locus, so the primary reference analysis used 27 unique loci.

Spatial non-randomness of IEL positions was evaluated by Monte Carlo placement of 34 unique centers among analytical alignment columns 1–2247. Two edge spacings (0.5 to the first center and the last center to 2247.5) were included; the coefficient of variation of the resulting 35 spacings and the maximum spacing were tested with 100,000 permutations, seed 20260716, and the +1 p-value correction. Separately, IEL–ECM overlap and ECM proximity to IEL boundaries were evaluated by interval-preserving permutation, with 27 unique *D. melanogaster* loci as the primary reference model and all 34 evolutionary IELs retained in sensitivity analysis; the stricter common circular-shift sensitivity model is described in the main Methods. The IEL passport and spatial-distribution tests are in Table S5, the primary IEL–ECM tests are summarized in Table S8, the projection diagram is in Figure S3, minimal data are in Data S2, and reproducibility code is in the public GitHub/Zenodo repository cited above.

SM5. Mobile Elements, Microsatellites, and ECMs

Candidate mobile-element traces were detected with CENSOR/Repbase and accepted only when position, orientation, and interspecific context were consistent with a large insertion. Microsatellites were used as the context of local length variation without a separate exhaustive enrichment analysis.

ECMs were discovered with MEME Suite 5.5.9. MEME was run on DNA under the ZOOPS model with both strands combined (-nmotifs 17, -minw 6, -maxw 30, -minsites 15, -maxsites 40, -markov_order 0). The search yielded 17 motifs of 29–30 nt supported by 24–37 primary sites; pairwise motif correlation did not exceed 0.60.

Formal matches were localized with MAST 5.5.9 using both strands. The individual-match threshold was $p \leq 1 \times 10^{-4}$ and the maximum sequence E-value was 10. MAST maps used unaligned species-specific sequences, so horizontal positions and line lengths were not treated as one shared interspecific coordinate scale. Match sign indicated forward or reverse-complement orientation only.

After orientation normalization and matching of repeated motif copies, the motifs were grouped into 17 ECM groups. The final three-species registry contained 49 occurrences: 17 in *D. melanogaster*, 17 in *D. yakuba*, and 15 in *D. virilis*. Table S3 contains search parameters, the final catalogue, and evidence summaries; raw MEME/MAST outputs are included in Data S3.

SM6. Final Agreed Functional-Object Registry

PAS, cleavage sites, UGUA, DSE, miRNA sites, ECMs, and MFE/centroid structural segments were harmonized for *D. melanogaster*, *D. yakuba*, and *D. virilis*. All intervals were represented in species coordinates and alignment3 and were assigned object type, orthology group, phyletic scope, final status (final/legacy/non-final), and QC flags.

The harmonized registry contains 1554 records: 1525 are in the final agreed set and 29 are retained as legacy records; 390 are primary functional objects and 1135 are structural segments. Summary inventories are in Table S6, the complete registry is in Data S3, and registry-validation code is in the public GitHub/Zenodo repository cited above.

SM7. Boundary Pair Universe and Boundary Neighborhoods

The Boundary Pair Universe was generated from pairwise-enabled QC-pass objects within each species. Duplicates and alternative MFE/centroid representations of the same interval were excluded. Immediate boundary neighborhoods were classified as SHARED_COORDINATE or ABUTTING_CUT; broader OVERLAP, TOUCHING, and SEPARATED relations were used only for geometric stratification.

Permutations were performed without replacement within strata preserving species, homotypy, layer relation, interval relation, and geometric bins. Homologous interfaces across the three focal species were defined by directed pairs of refined orthology groups. Method summaries are in Table S7, compact data are in Data S3, and the BPU builder, stratified permutation procedure, and validation code are in the public GitHub/Zenodo repository cited above.

SM8. Coverage Enrichment and Functional Distance

Both analyses used the final agreed set of 510 *D. melanogaster* objects within coordinates 1–964. Coverage Enrichment compared covered-nucleotide fractions among structural classes, individual ECMs, pooled ECMs, and APA-centered windows. Before randomization, overlapping or adjacent feature intervals were merged; the number and multiset of merged-interval lengths were preserved, and intervals were relocated independently within the 964-nt 3′ UTR. Randomized intervals could overlap, whereas tested and control region masks remained fixed.

Functional Distance used PAS and cleavage sites as fixed landmarks. The primary statistic was the median minimum edge-to-edge distance; self-comparisons were excluded. Both modules used 10,000 permutations and seed 20260718; BH/FDR was calculated within predefined families. Full results are in Table S9, machine-readable output is in Data S4, and reproducibility code is in the public GitHub/Zenodo repository cited above.

SM9. PAT, AEM, and the Basal Architecture Network

The Pairwise Architecture Test evaluated ECM–predicted RNA structure, ECM–APA, and predicted RNA structure–APA using the final agreed set of 49 ECM occurrences, 127 APA objects, and 1135 predicted structural segments. The null model comprised the complete set of 1314 non-zero circular shifts of alignment3.

The Architecture Evidence Matrix was rebuilt for 34 IELs × three species. The BAN was used as an evidence network of four layers—IEL, ECM, predicted RNA structure, and APA. Each edge retained its evidence source and support grade and was not interpreted as direct three-dimensional contact. Results are in Table S8 and Figure S4; machine-readable output is in Data S4, and reproducibility code is in the public GitHub/Zenodo repository cited above.

SM10. Between-Context Dispersion of Cleavage-Site Usage

The tissue-resolved dataset contained 29 cleavage sites and five comparable contexts per site: head, ovary, testis, embryo 0–12 h, and embryo 12–24 h. Final five-component consensus structural profiles from centroid and MFE annotations were available for 27 sites. Usage fractions were transformed to empirical logits with a 0.5 correction and centered on the mean of each site.

Weighted variance of centered values was calculated for each state. Complete five-component profiles were permuted among sites within species while the five contexts of each site remained together. The primary DIFFERENT_MULTILOOP_ARMS − SAME_MULTILOOP_INTERVAL contrast was tested with 100,000 permutations and seed 20260727. Additional analyses covered adult tissues, embryonic stages, centroid/MFE models, alternative pair scopes, species-specific tests, and leave-one-species-out sensitivity.

Because the dataset contains one aggregated observation for each species × tissue/stage combination, the analysis estimates between-context heterogeneity rather than biological replicate variance within tissues. Compact results are in Table S10 and Figure S5, the full technical package is in Data S5, and reproducibility code is in the public GitHub/Zenodo repository cited above.

Final Supplementary Composition

The submission folder contains Supplementary Methods SM1–SM10, Supplementary Results SR1–SR7, Tables S1–S10, Figures S1–S5, Data S1–S5, and the Supplementary Master Index. Analysis code is available in the public GitHub repository https://github.com/alexgenego/ras85d-3utr-architecture and in the permanently archived manuscript-associated Zenodo release v1.0.0 (https://doi.org/10.5281/zenodo.22108845). The Supplementary Master Index provides the complete scientific correspondence and consolidated QC status; the final file manifest records package paths, file sizes, SHA-256 checksums, and presence status.

Quality-Control Summary

Component	Primary check	Final status
Sequences and coordinates	37 taxa; 2276/2247 columns; 36 single-scaffold + 1 split-scaffold mapping	PASS
Phylogeny and ARPIP	canonical N01–N34; 63 positive-length ingroup branches	PASS
Functional architecture	1525 final-analysis records; 49 ECM occurrences; 1135 structural segments	PASS
Permutation analyses	fixed seeds and permutation counts; BH/FDR within predefined families	PASS
PAT/AEM; Coverage/Distance	final reruns completed; historical input discrepancies resolved; final QC passed	PASS

Availability of Extended Data and Code

The compact Supplementary package contains the data, tables, and figures required to understand and reproduce the analyses reported here. Analysis scripts and reproducibility code are available in the public GitHub repository https://github.com/alexgenego/ras85d-3utr-architecture and in the permanently archived Zenodo release v1.0.0 (https://doi.org/10.5281/zenodo.22108845). Extended diagnostic tables, full execution logs, intermediate and legacy analysis versions, additional sensitivity results, and large machine-readable evidence matrices are retained in the internal project archive and are available from the corresponding author upon reasonable request.
