# Supplementary material for: The Evolutionary Plasticity, Conservation of Functional Motifs, and Structural—Functional Architecture of the ras85D 3′ UTR in Drosophila
Source: Genes (Basel). 2026 Aug 29;17(9):1041. doi: 10.3390/genes17091041 (PMC13606288; doi:10.3390/genes17091041)
Supplement: Supplementary file 1 [file genes-17-01041-s001.zip › 02_Results/Supplementary_Results_EN.pdf]

Supplementary Results

Key Additional Results and Evidence

Overview

The Supplementary Results include only the evidence needed to verify the principal conclusions of the manuscript. Complete values are provided in Tables S1–S10; Figures S1–S5 provide additional visual checks. Technical and private sensitivity results are not duplicated.

SR1. Sources, Alignment, and RelTime

After correction of five technical taxon labels, the FASTA and tree taxon sets were identical. All 37 sequences had confirmed mapping; *D. borealis* was the only split-scaffold sequence. The analytical matrix contained 2247 of the 2276 original columns (Table S1; Data S1).

Median terminal relative rates were 0.597 under Partial Deletion and 0.615 under Complete Deletion; no systematic shift was detected ($p = 0.1668$), and taxon-specific agreement was high. Cumulative root-to-tip rates were higher under Partial Deletion for all 36 taxa. The strict clock was rejected for both matrices. Full values are in Table S2 and Figure S1.

SR2. ARPIP, Branch Regimes, and IELs

ARPIP reconstruction produced 959 block events: 249 insertions and 710 deletions. In the 63-branch ingroup universe, one branch showed bidirectional turnover, three were insertion-enriched, 16 were deletion-enriched, and 43 were non-significant. Stable IDs and final regimes are shown in Table S4 and Figure S2.

Thirty-four IELs occupied 123 of 2247 alignment columns and projected to 27 unique *D. melanogaster* loci. Their spatial distribution differed from random expectation for both the coefficient of variation of spacing ($p = 0.00698$) and the maximum gap ($p = 0.01079$). Details are in Table S5 and Figure S3.

SR3. Mobile Elements, Microsatellites, and ECMs

Three candidate mobile-element traces were accepted: two Gypsy-18_DVir-I and one Helitron-2_DSuz. They are interpreted as lineage-associated insertion traces rather than evidence of intact conserved transposons. Microsatellites are treated as context for local length variation. Candidate mobile-element and microsatellite evidence is summarized in Table S3.

MEME identified 17 ECM models of 29–30 nt. Each model was supported by 24–37 primary sites, with E-values from 7.3×10^{-399} to 9.9×10^{-132}. MAST detected combined motif signatures in all sequences of the original dataset; combined p-values ranged from 3.1×10^{-188} to 2.05×10^{-29}. Relative motif order was more stable than inter-motif spacing, whereas *D. willistoni*, *S. lebanonensis*, *D. busckii*, *D. albomicans*, and *D. grimshawi* showed the strongest changes in intervals, additional matches, and orientation.

The final three-species registry contains 49 occurrences in 17 ECM groups: 17 in *D. melanogaster*, 17 in *D. yakuba*, and 15 in *D. virilis*. *D. virilis* lacks ECM16 and ECM17, and ECM11 occurs in reverse-complement orientation. Species coordinates and orientations are in Table S3; raw MEME/MAST outputs are in Data S3.

Main Figure 2 shows formal MAST locations in unaligned species-specific sequences. Differences in line length and horizontal position are therefore used to compare motif composition, order, and local copies but not as one shared coordinate scale.

SR4. Functional Registry and Boundary Neighborhoods

Final harmonization yielded 1554 records: 1525 entered the final agreed analysis set and 29 were retained as legacy records. The set contains 390 primary functional objects and 1135 structural segments, with no critical QC errors. The registry and interspecific summary are in Table S6 and Data S3.

The Boundary Pair Universe contained 137,550 possible pairs and 107,321 eligible pairs; 871 observed pair relations across all architectural layers were mapped to the universe. Within the strict functional-interface subset, 109 interfaces were represented by 78 homologous interface families. Of these families, 30 occurred in at least two species and 48 were species-specific. The only boundary-neighborhood type change involved PAS1.5→ECM_2: ABUTTING_CUT in *D. yakuba* and SHARED_COORDINATE in *D. virilis*; its exact branch placement is ambiguous (Table S7).

SR5. Coverage Enrichment and Functional Distance

Coverage Enrichment generated 1414 tests. Six rows remained significant after FDR correction; four were internal positive controls for APA-centered windows. Two independent results showed no weak-miRNA-covered nucleotide in ECM_15 (1–30) or ECM_11 (230–258) relative to non-ECM sequence; both had FDR = 0.019548.

Functional Distance comprised 12 tests to PAS and cleavage sites; none was nominally or FDR-significant. The preliminary conserved-miRNA→PAS tendency was not supported by the final agreed annotation of six conserved sites ($p = 0.2982$; FDR = 0.5963; Table S9).

SR6. PAT, AEM, and the Integrated BAN

PAT on the final agreed set generated 43 unique metrics. Predicted RNA structure–APA was the only FDR-significant primary edge: observed boundary-to-center = 22.272727, null mean = 4.272117, O/E = 5.213510, $p = 0.003802$, and FDR = 0.007605. ECM–APA and ECM–predicted RNA structure did not reach FDR significance.

AEM resolved all 34 IELs: 14 conserved-domain and 20 partially conserved-domain classes. In the final BAN, IEL–ECM and predicted RNA structure–APA are FDR-supported; IEL–predicted RNA structure is retained as supported by structural-boundary preference, whereas the other edges have nominal or negative status (Table S8; Figure S4).

SR7. Between-Context Dispersion of APA Structural States

DIFFERENT_MULTILOOP_ARMS did not show absolute excess variance relative to its own null: observed/null = 1.090, $p_{high} = 0.161408$, and FDR = 0.807042. SAME_MULTILOOP_INTERVAL instead showed significantly reduced variance: observed/null = 0.276, $p_{two} = 0.004660$, and FDR = 0.023300.

The primary DIFFERENT_MULTILOOP_ARMS − SAME_MULTILOOP_INTERVAL contrast was 0.777288 ($p = 0.001490$; FDR = 0.004470). It persisted in the three adult tissues (FDR = 0.027570) and the early-versus-late embryo comparison (FDR = 0.030910). Direction was positive in all three species and all leave-one-species-out analyses.

The result was supported by consensus, centroid, component-only, and cleavage-involving analyses, whereas the MFE-only test was not significant. The conclusion is therefore relative regulatory plasticity: co-location within one multiloop interval is associated with constrained between-context variation, whereas different arms permit a broader range of tissue- and stage-dependent states.

Supplementary Figure Captions

Figure S1. Sensitivity of RelTime estimates to alignment-site filtering. Panel A presents the Complete Deletion terminal relative-rate profile; panels B–E compare Partial and Complete Deletion terminal and cumulative root-to-tip rates.

Figure S2. Canonical phylogeny with stable internal IDs N01–N34 and final branch-specific indel regimes. The outgroup and zero-length branches are retained for topology but excluded from the 63-branch statistical universe.

Figure S3. Distribution of 34 IELs across the 2247-column analytical alignment and their projection to 27 unique *D. melanogaster* loci. Symbols indicate projection class.

Figure S4. Four-node evidence network integrating IEL, ECM, predicted RNA secondary structure, and APA. Edge grades preserve the strength and provenance of the underlying tests and do not imply direct three-dimensional molecular contact.

Figure S5. Between-context dispersion of cleavage-site usage in five APA-component structural states. (A) Observed weighted variances and 95% intervals of null distributions. (B) DIFFERENT_MULTILOOP_ARMS − SAME_MULTILOOP_INTERVAL contrasts for all five contexts, adult tissues, and embryonic stages. (C) Species-specific direction of the contrast.

Evidence Summary

Supplementary block	Key result	Evidence
SR1 / SM1	37 taxa; 2276 columns; analytical columns 1–2247; 36 single + 1 split mapping	Table S1; Data S1
SR1 / SM2	Partial and Complete Deletion give concordant terminal rates; strict clock rejected	Table S2; Figure S1
SR2 / SM3–SM4	959 indel events; 63 branches; 34 IELs; 27 unique *D. melanogaster* loci	Tables S4–S5; Figures S2–S3; Data S2
SR3 / SM5	3 mobile-element candidates; 17 ECM groups; 49 three-species ECM occurrences	Table S3; Data S3; main Figure 2
SR4 / SM6	1525 final-analysis records; 390 primary objects; 1135 structural segments	Table S6; Data S3
SR4 / SM7	107,321 eligible BPU pairs; 109 strict functional interfaces; 78 homologous families; one type change	Table S7; Data S3
SR5 / SM8	1414 coverage tests: two independent FDR findings; 12 distance tests: none significant	Table S9; Data S4
SR6 / SM9	Predicted RNA structure–APA: O/E = 5.2135, FDR = 0.007605; 14 conserved + 20 partial AEM classes	Table S8; Figure S4; Data S4
SR7 / SM10	Reduced SAME_MULTILOOP_INTERVAL variance and significant different-arms contrast	Table S10; Figure S5; Data S5
